# Memory B cell response to SARS-CoV-2 vaccination in people with heart and lung transplantation: Identification of associated clinical factors

**DOI:** 10.1016/j.isci.2026.117428

**Published:** 2026-09-07

**Authors:** Chiara Coppola, Simone Costagli, Giorgio Montesi, Simone Lucchesi, Jacopo Polvere, Arianna Lippi, Fabio Pierguidi, Francesca Montagnani, Massimiliano Fabbiani, Sonia Bernazzali, David Bennett, Donata Medaglini, Annalisa Ciabattini

**Affiliations:** 1 Laboratory of Molecular Microbiology and Biotechnology, Department of Medical Biotechnologies, University of Siena, Siena, Italy; 2 Department of Medical Sciences, Infectious and Tropical Diseases Unit, University Hospital of Siena, Siena, Italy; 3 Department of Medical Biotechnologies, University of Siena, Siena, Italy; 4 Department of Cardiac Surgery, University of Siena, Siena, Italy; 5 Respiratory Disease and Lung Transplant Unit, University of Siena, Siena, Italy; 6 Mycobacteriology and Cell Response to Infection Programme, Department of Cell Therapies, Hematology and Laboratory Medicine, University Hospital of Siena, Siena, Italy

**Keywords:** solid organ transplant recipients, clustering analysis, SARS-CoV-2 vaccination, omicron-adapted vaccines, memory B cell profiling

## Abstract

Solid organ transplant recipients (SOT) have shown impaired immune responses to infection and vaccination. Here, serological data collected in heart and lung transplant recipients after vaccination against SARS-CoV-2 were analyzed using dimensionality reduction, clustering algorithms and statistical methods to define factors associated with immune responsiveness. Transplanted organ type, bivalent Omicron-adapted vaccine administration, and immunosuppressive regimen were associated with distinct serological profiles. These factors were associated with significant differences in the RBD-specific memory B cell (MBC) responses. Indeed, a lower frequency of MBC recognizing both wild-type and Omicron BA.2 RBD was detected in lung versus heart transplant recipients. Administration of the bivalent Omicron-adapted fifth dose was associated with the modulation of RBD-specific MBC phenotypes, and the mycophenolate mofetil (MMF) treatment was associated with significantly lower humoral and MBC responses in heart transplant recipients. This work identifies factors associated with vaccine-induced immune responses in SOT recipients, useful to inform tailored vaccination strategies.

## Highlights

• SARS-CoV-2 vaccine-induced immune responses differ by organ transplant type
• RBD-specific B cell subsets modulation upon Omicron-adapted vaccination
• Reduced humoral and B-cell responses in heart transplant recipients on MMF treatment

## Graphical abstract

**Figure:**
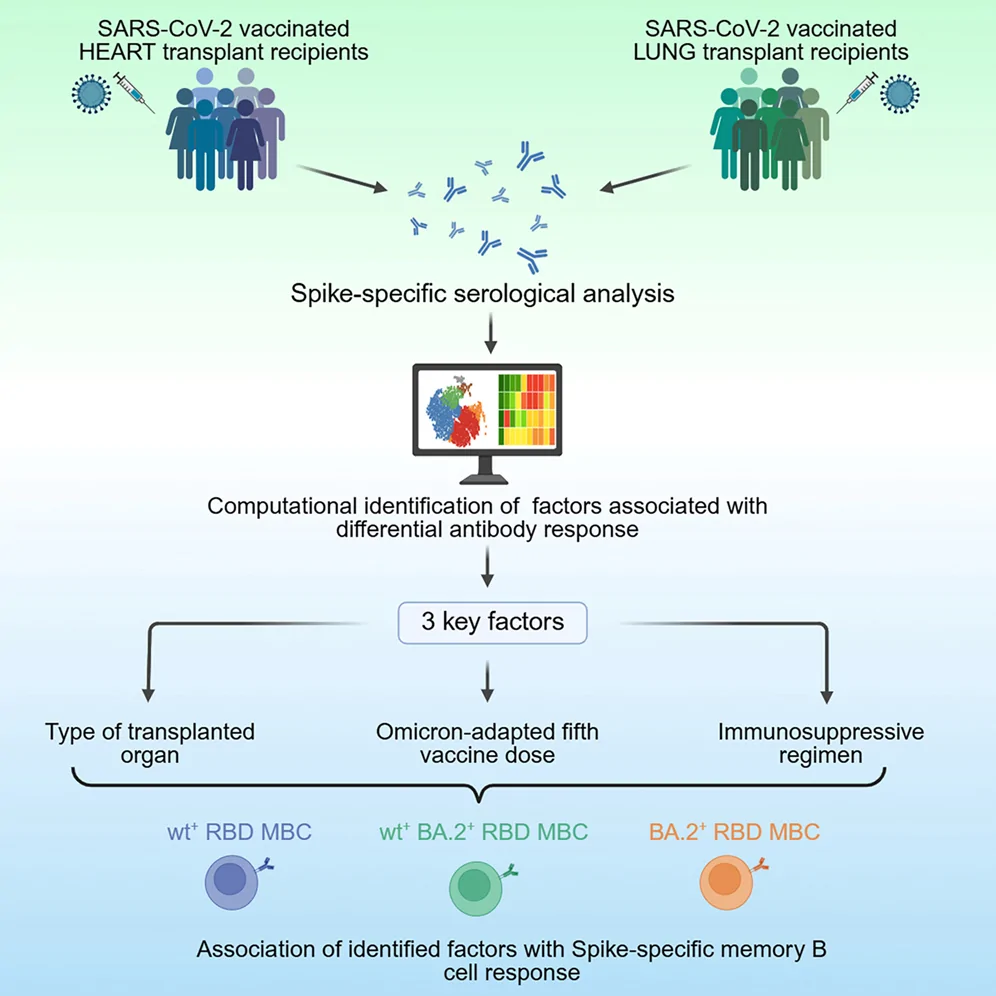

## Teaser

Health sciences; Medicine; Medical specialty; Immunology

## Introduction

Solid organ transplant (SOT) recipients are a group of immunocompromised patients who, due to the immunosuppressive therapy and the complexity of their clinical condition, often exhibit a reduced ability to develop an effective immune response to infections^1^^,^^2^^,^^3^ and vaccinations.^4^^,^^5^^,^^6^^,^^7^^,^^8^

In the context of the SARS-CoV-2 pandemic, several studies have investigated the immune responses induced in SOT recipients by the wild-type (wt) and Omicron-adapted vaccines against SARS-CoV-2. These studies have highlighted suboptimal immunogenicity^9^^,^^10^^,^^11^ compared to healthy individuals, while also demonstrating a protective effect of vaccination from the severe forms of COVID-19.^12^^,^^13^^,^^14^

However, while antibody response has been deeply studied in this population, less is known about the memory B cell (MBC) response. MBC have a crucial role for providing long lasting immunity and protection, since they are capable of rapid reactivation following secondary encounters with the pathogen, differentiating into antibody-secreting plasma cells, or re-entering into the germinal center (GC), where they increase their affinity through several rounds of affinity selection.^15^ Characterization of the MBC response directed against SARS-CoV-2 in immunocompromised individuals can yield critical insights into the state of immune memory established after repeated vaccination against a rapidly evolving pathogen, informing immunization strategies for future epidemic or pandemic pathogens.

During the SARS-CoV-2 pandemic, computational approaches have been increasingly applied to the integrative analysis of high-dimensional immunological data, enabling the identification of clinical variables influencing vaccine responsiveness among specific groups of vaccinees,^16^^,^^17^^,^^18^ the characterization of transcriptomic signatures predictive of immune response^19^^,^^20^^,^^21^ and the dissection of hybrid immunity.^22^ In the context of future epidemic challenges, these analytical approaches may prove highly valuable for rapidly and efficiently exploring complex immunological datasets, providing critical insights to guide vaccination protocols. This can be particularly relevant for immunocompromised individuals such as SOT recipients, who have an increased need for timely and effective immunization strategies.

In this study, serological data collected two years after the first dose from heart and lung transplant recipients were analyzed using dimensionality reduction and clustering algorithms to derive a data-driven classification of each individual antibody response. Immune responses were assessed against both wt and Omicron BA.2 spike antigens. The Omicron BA.2 viral variant was selected as representative of the Omicron lineage, given the predominance of closely related sublineages at the time of blood sampling. Based on the resulting cluster distribution, relevant factors associated with distinct antibody response profiles were identified, and their association with the RBD-specific MBC response was subsequently evaluated.

## Results

### Participants

**Figure 1:**
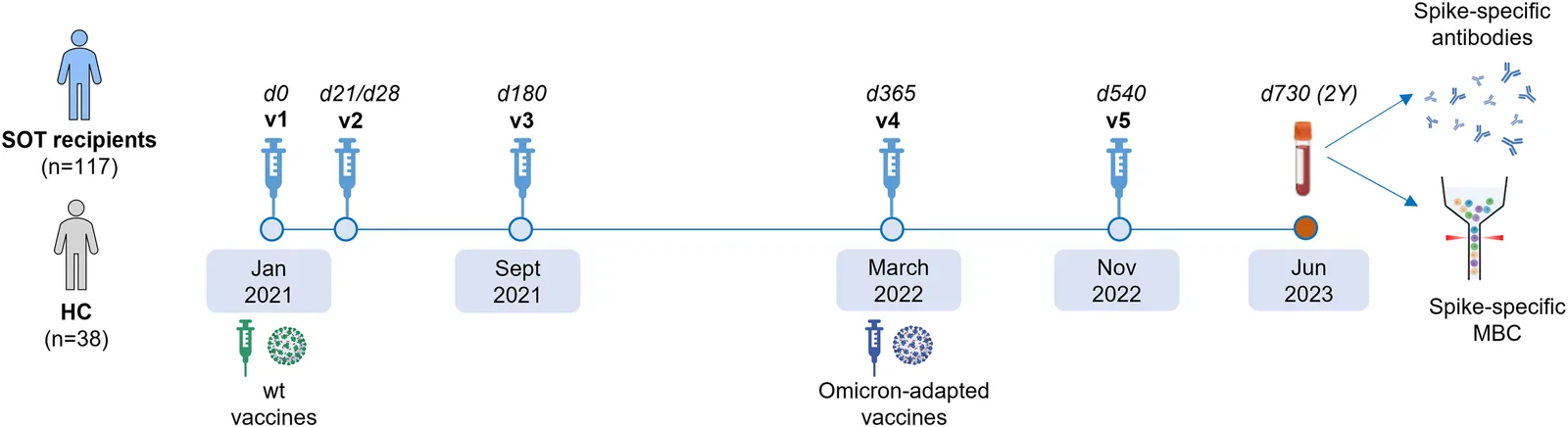
Study design A total of 117 SOT recipients and 38 HC vaccinated against SARS-CoV2 with mRNA vaccines were enrolled in the study. mRNA vaccination was performed at day 0 (v1), 21–28 days (v2), 180 days (v3), 365 days (v4) and 540 days (v5) from the first vaccine dose. The emergence of Omicron variants occurred between v3 and v4. Monovalent wt SARS-CoV-2 vaccines were used at v1-v4. Bivalent Omicron-adapted vaccines were introduced at v4 and became the only formulation at v5. Blood samples were collected two years after the first vaccine dose (in a range of time between May to September 2023). Plasma and PBMC were separated from blood samples and assessed for spike-specific humoral and cellular B responses. SOT recipients, solid organ transplant recipients; HC, healthy controls.

The spike-specific antibody and B cell responses were analyzed in 117 SOT recipients and 38 healthy controls (HC), two years after the first dose. The study was conducted from May to September 2023. Subjects were enrolled in the PatoVac_COV and IMMUNO_COV longitudinal studies, respectively, conducted at the Siena University Hospital (Siena, Italy), since the beginning of the SARS-CoV-2 vaccination campaign (Figure 1). Given the high proportion of SOT recipients who received four or more vaccine doses, only HC with a history of four or more antigenic exposures (through vaccination and/or infection) were included in the analysis. Demographic, clinical, SARS-CoV-2 vaccination, and infection data are summarized in Table 1. SOT recipients’ group included recipients of heart (HTx recipients; *n* = 80, 68.38%) and lung (LuTx recipients; *n* = 37, 31.62%) transplantation. Among the HTx and LuTx recipient groups, 64 (80%) and 18 (48.65%) were male, respectively. The median age at the time of the first dose was 63 years (interquartile range, IQR 55–68) in the HTx recipient group, and 56 years (IQR 47–61) in the LuTx recipient group. Median time since transplantation was 10 years (IQR 6–15) in the HTx recipient group and 5 years (IQR 1–7) in the LuTx recipient group. The HC group was mainly populated by female individuals (*n* = 24, 63.16%) with a median age of 49 years (IQR 43–57).

**Table 1:** Description of the SOT recipient (*n* = 117) and HC (*n* = 38) groups

	HTx recipients (*n* = 80; 68.38%)	LuTx recipients (*n* = 37; 31.62%)	HC (*n* = 38)
Sex at birth			
Female – n (%)	16 (20)	19 (51.35)	24 (63.16)
Male – n (%)	64 (80)	18 (48.65)	14 (36.84)
Median age^a^ – IQR	63 (55–68)	56 (47–61)	49 (43–57)
Median time since transplant^b^ – (IQR)	10 (6–15)	5 (1–7)	/
Acute organ rejection since 2020			
Grade A1	0 (0)	5 (13.51)	/
Grade A2 or higher	0 (0)	3 (8.11)	/
Chronic organ rejection			
Treated with photopheresis - n (%)	0 (0)	1 (2.70)	/
Not treated with photopheresis - n (%)	0 (0)	2 (5.40)	/
Vaccine 1^st^ and 2^nd^ doses (wt) - n (%)	80 (100)	37 (100)	38 (100)
Vaccine 3^rd^ dose (wt) - n (%)	78 (97.50)	36 (97.30)	38 (100)
Vaccine 4^th^ dose			
wt - n (%)	54 (67.50)	30 (81.08)	11 (28.95)
Bivalent, Omicron adapted - n (%)	9 (11.25)	4 (10.81)	2 (5.26)
Not received – n (%)	16 (20)	3 (8.11)	25 (65.79)
Vaccine 5^th^ dose			
Bivalent, Omicron adapted - n (%)	30 (37.50)	14 (37.84)	0 (0)
Not received – n (%)	50 (62.50)	23 (62.16)	38 (100)
Previous Infection – n (%)			
Pre-Omicron era^c^	3 (3.75)	3 (8.11)	2 (5.26)
Omicron era	45 (56.25)	17 (45.94)	30 (78.94)

SOT recipients, solid organ transplant recipients; HC, healthy controls; LuTx recipients, lung transplant recipients; HTx recipients, heart transplant recipients.

a The median age was calculated at the time of the first vaccine dose received.

b The median time since transplant, in years, was calculated at the time of sample collection (2023).

c Before 27th November 2021 (https://www.epicentro.iss.it).

All participants were vaccinated with mRNA vaccines. A small fraction of participants received a bivalent Omicron-adapted vaccine formulation (wt/Omicron BA.1 or wt/Omicron BA.4-5) as their fourth dose, corresponding to 12.50% of HTx recipients, 10.81% of LuTx recipients, and 5.26% of HC. Subsequently, all HTx and LuTx recipients who received the fifth dose, administered between October and December 2022, were boosted with a bivalent Omicron-adapted vaccine formulation, corresponding to 37.50% of HTx recipients and 37.84% of LuTx recipients.

Within the HTx recipient group, participants were undergoing a low (*n* = 6, 7.5%), medium (*n* = 41, 51.25%) and high (*n* = 33, 41.25%) immunosuppressive regimen, as reported in Table 2. Most of them included in their treatment tacrolimus (*n* = 52, 65%), prednisone (*n* = 43, 53.75%) and/or mycophenolate mofetil (*n* = 41, MMF, 51.25%). All LuTx recipients were treated with prednisone and tacrolimus, and in some cases also with MMF (*n* = 22, 59.46%), or everolimus (*n* = 8; 21.62%).

**Table 2:** Immunosuppressive regimens among HTx and LuTx recipients

Immunosuppressive regimen	HTx recipients (*n* = 80)	LuTx recipients (*n* = 37)
Low intensity (single drug regimen)	6 (7.50%)	0 (0%)
Medium intensity (2-drug regimen)	41 (51.25%)	7 (18.92%)
High intensity (3-drug regimen)	33 (41.25%)	30 (81.08%)
Prednisone included	43 (53.75%)	37 (100%)
Cyclosporine included	21 (26.25%)	0 (0%)
Tacrolimus included	52 (65.00%)	37 (100%)
MMF included	41 (51.25%)	22 (59.46%)
Everolimus included	30 (37.5%)	8 (21.62%)

LuTx recipients, lung transplant recipients; HTx recipients, heart transplant recipients; MMF, mycophenolate mofetil.

### Characterization of spike-specific humoral response in SOT recipients

**Figure 2:**
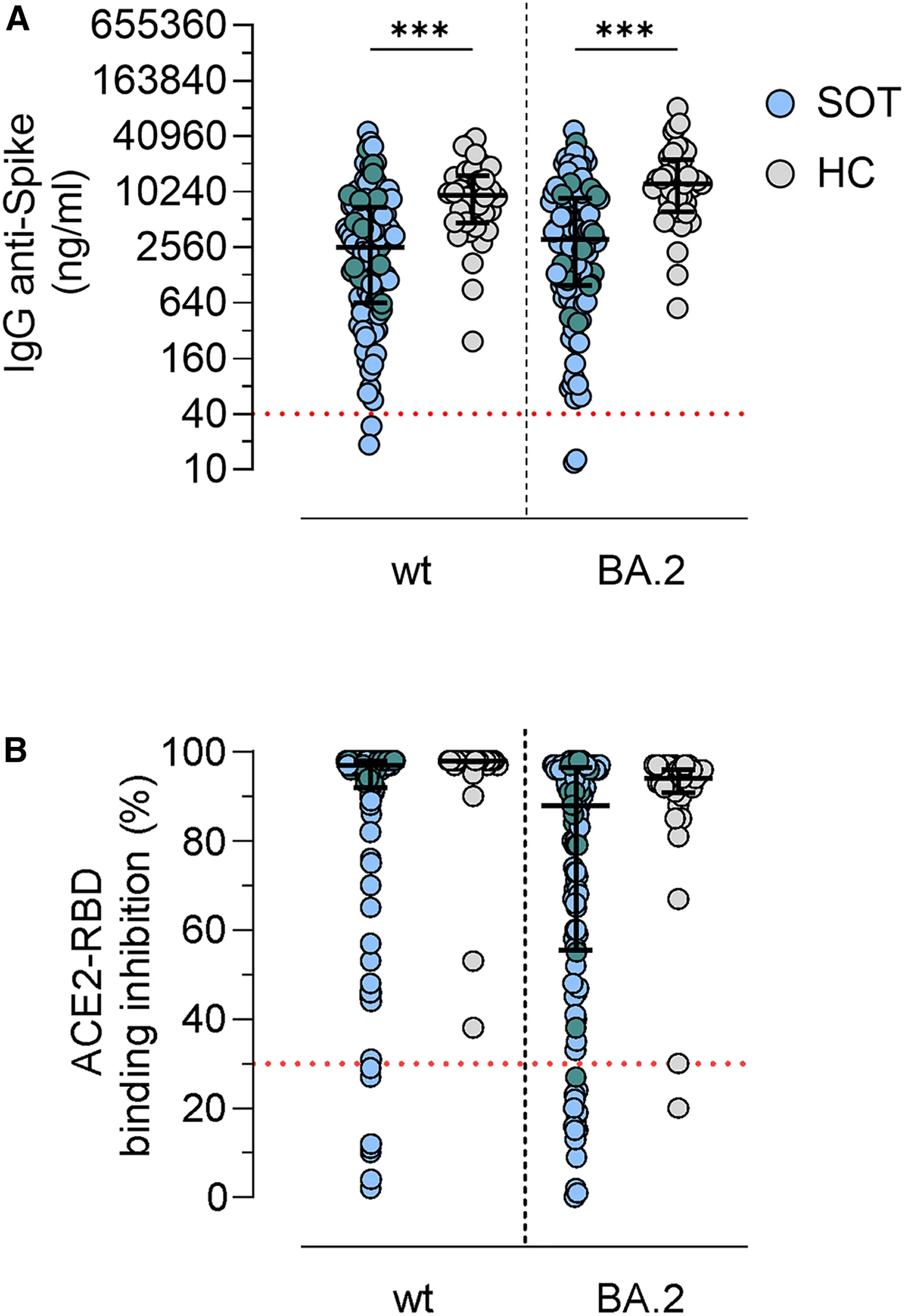
Antibody response in SOT recipients (A) Spike-specific IgG targeting the wt strain and the Omicron BA.2 variant (BA.2) were assessed in the solid organ transplant recipients (SOT) and healthy controls (HC) 2 years after the first vaccine dose. Values are reported as antibody concentration (ng/mL). A threshold (dotted red line) was placed at the pre-pandemic cut-off for wt spike-specific IgG to discriminate between seropositive and seronegative samples. (B) Antibody capacity to inhibit the ACE2/RBD binding for the wt strain and the Omicron BA.2 variant, assessed by sVNT. A threshold (dotted red line) was placed at 30% inhibition percentage to discriminate between positive and negative samples. SOT recipients who had received SARS-CoV-2 vaccination prior to organ transplantation are highlighted in dark green. ∗∗∗*p* < 0.001. Sample size: SOT (A-B: *n* = 117); HC (A-B: *n* = 38).

The SOT recipient group showed significantly lower levels of wt and Omicron BA.2 spike-specific IgG antibodies compared to HC, two years after the first dose (∗∗∗*p* < 0.001, H Kruskal-Wallis value = 56.54, Figure 2A). The functionality of spike-specific antibodies was assessed by employing a SARS-CoV-2 surrogate virus neutralization test (sVNT), which evaluates the antibody capacity to bind the RBD, thus blocking its interaction with the ACE2 receptor. Most SOT recipients exhibited antibodies with ACE2/RBD binding inhibition capacity above the threshold (>30%) for both wt (94% of the SOT recipients group) and BA.2 RBD antigens (85% of the SOT recipients group). These frequencies were slightly lower compared to those of HC (100% and 97% of HC for the wt and BA.2 antigens, respectively) (Figure 2B). The observed differences were not driven by age or sex distribution, as demonstrated by computational subsampling matching SOT recipients and HC for these variables (Figure S1).

### Unsupervised clustering identifies SOT recipient subgroups with distinct spike-specific antibody and memory B cell responses

Unsupervised clustering was employed to dissect inter-individual heterogeneity in SARS-CoV-2–specific humoral immunity within the SOT recipient group, based on IgG concentrations and ACE2/RBD binding inhibition capacity against wt and Omicron BA.2. A detailed description of the unsupervised clustering analysis is reported in supplemental information (Figures S2 and S3; Tables S1 and S2; Methods S1). The four serological features were subjected to k-means and Gaussian mixture modeling (GMM) in four data representations: the original 4-dimensional space and three 2-dimensional embeddings generated by PCA, t-SNE, and UMAP, yielding eight distinct clustering workflows (Figure S2). All strategies were benchmarked by computing Within-Cluster Sum of Squares (WCSS) and Average Silhouette Width (ASW; Table S1 and Figure S2). Of the two solutions with the highest ASW—k-means on PCA-reduced space and k-means on UMAP—the latter achieved a lower WSS (367 versus 385), indicating greater intra-cluster compactness. Given these results, the k-means-UMAP approach was selected for downstream analyses.

**Figure 3:**
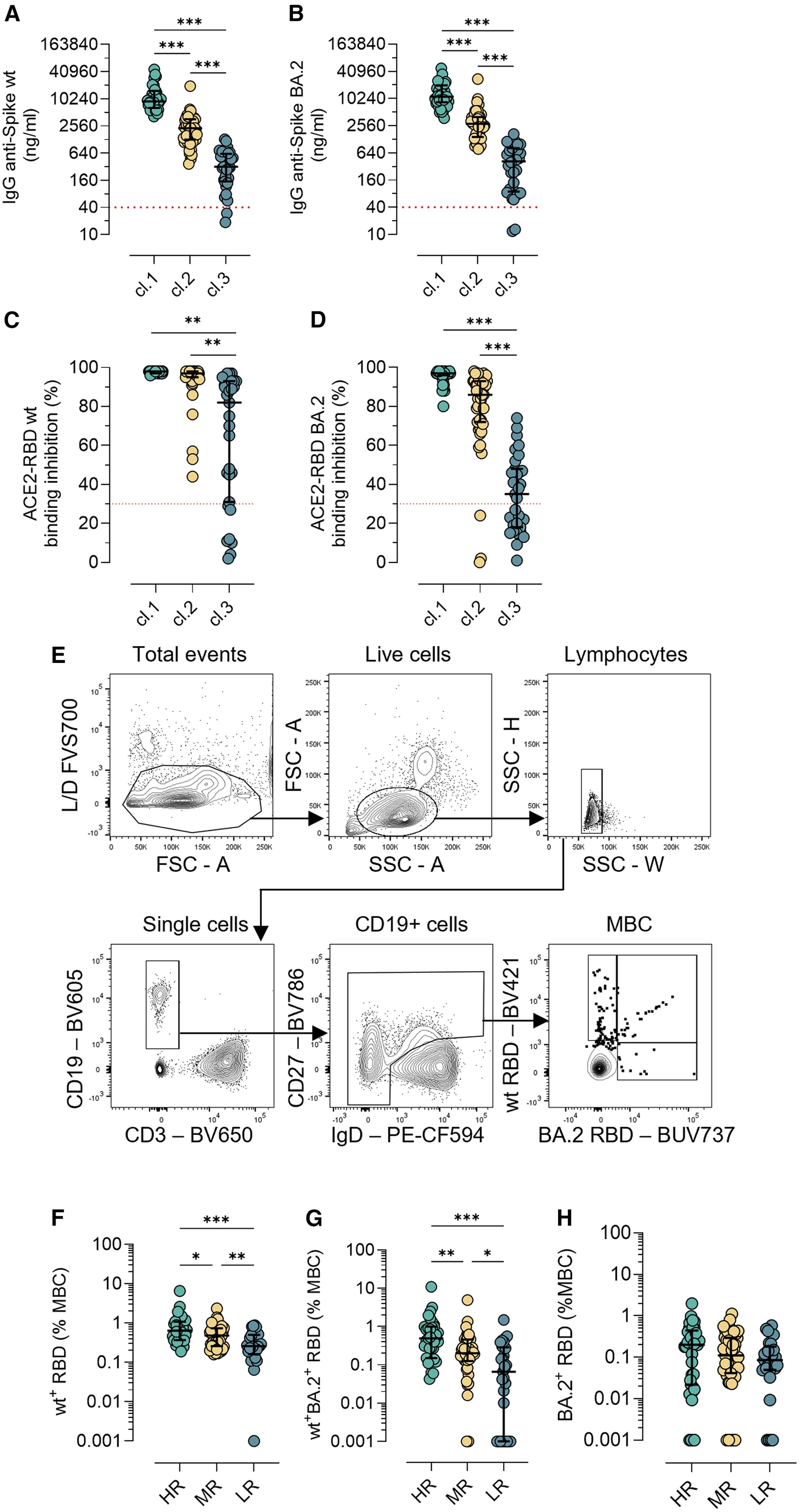
Immune response across SOT recipient clusters of responders (A and B) Spike-specific IgG targeting the wt strain (A) and the Omicron BA.2 variant (B), assessed in the cluster 1 (cl. 1), cluster 2 (cl. 2) and cluster 3 (cl. 3). Values are reported as antibody concentration (ng/mL). A threshold (dotted red line) was placed at the pre-pandemic cut-off for wt spike-specific IgG to discriminate between seropositive and seronegative samples. (C and D) ACE2/RBD binding inhibition capacity of plasma antibodies, assessed against the wt strain (C) and the Omicron BA.2 variant (D), analyzed in the cluster 1 (cl. 1), cluster 2 (cl. 2) and cluster 3 (cl. 3). A threshold (dotted red line) was placed at 30% inhibition percentage to discriminate between positive and negative samples. (E) Gating strategy for identifying wt^+^ RBD, wt^+^BA.2^+^ and BA.2^+^ RBD MBC by multiparametric flow cytometry (representative dot plot). (F–H) Frequency of wt^+^ RBD (F), wt^+^BA.2^+^ (G) and BA.2^+^ RBD MBC (H) assessed among the SOT recipient clusters of responders. Data (A–D and (F–H) are shown as dot plots, with the median and error bars representing the interquartile range. Kruskal-Wallis test, followed by Dunn’s post-test for multiple comparisons, was used for assessing statistical differences between groups in (A–B) and in (F–H). Fisher’s exact test was used for assessing statistical differences in the number of participants with antibodies with positive ACE2/RBD binding inhibition value in (C–D). ∗*p* < 0.05, ∗∗*p* < 0.01, ∗∗∗*p* < 0.001. Sample size: HR (A–D: *n* = 39; F-H: *n* = 35); MR (A–D: *n* = 47; F-H: *n* = 45); LR (A–D: *n* = 31; F-H: *n* = 29). HR = high responders; MR = medium responders; LR = low responders.

This strategy identified 3 clusters of immune response profiles within the SOT recipient group, grouping individuals based on similar antibody levels and ACE2/RBD binding inhibition (Figure S3). Cluster 1 exhibited significantly higher wt and Omicron BA.2 spike-specific IgG responses compared to cluster 2 and 3 (both ∗∗∗*p* < 0.001, H Kruskal-Wallis values = 94.84 and 92.60 respectively, Figures 3A and 3B) and a significantly higher frequency of participants developing antibodies with ACE2/RBD binding inhibition capacity above the threshold compared to cluster 3 (∗∗*p* < 0.01 and ∗∗∗*p* < 0.001, respectively, Figures 3C and 3D). In contrast, cluster 3 displayed markedly reduced wt and BA.2 spike-specific IgG levels compared to clusters 1 and 2 (both ∗∗∗*p* < 0.001), and two participants did not mount a spike-specific IgG response at all, showing wt and BA.2 spike-specific antibody levels below the pre-pandemic baseline cut-off. Additionally, the frequency of participants developing ACE2/RBD binding inhibition capacities above the threshold was significantly lower compared to clusters 1 and 2 (both ∗∗*p* < 0.01 for ACE2/wt RBD binding inhibition capacities; both ∗∗∗*p* < 0.001 for ACE2/BA.2 RBD binding inhibition capacities Figures 3A–3D). Consequently, cluster 1 is hereafter referred to as high responders (HR, *n* = 39 subjects), cluster 2 as medium responders (MR, *n* = 47 subjects), cluster 3 as low responders (LR, *n* = 31 subjects).

The high, medium, and low responder groups, identified based on serological data were further evaluated for their RBD-specific MBC responses. RBD-specific MBCs were identified among CD19^+^ cells, after exclusion of the IgD^+^ CD27^−^- naiïve population in PBMC samples (Figure 3E). B cells reactive against the wt RBD, the BA.2 RBD variant, or both were identified using fluorescent WT and BA.2 RBD antigens. The flow cytometry analysis highlighted subsets of MBC recognizing wt-specific epitopes (wt^+^ RBD MBC), BA.2-specific epitopes (BA.2^+^ RBD MBC), or conserved epitopes across wt and BA.2 viral variants (wt^+^BA.2^+^ RBD MBC; Figure 3E). HR displayed significantly higher frequencies of both wt^+^ RBD MBC and wt^+^BA.2^+^ RBD MBC compared to MR (∗*p* = 0.028 and ∗∗*p* = 0.004, H Kruskal-Wallis values = 19.80 and 24.49, respectively) and LR (both ∗∗∗*p* < 0.001, Figures 3F and 3G). LR displayed the lowest levels of both wt^+^ RBD MBC and wt^+^BA.2^+^ RBD MBC among the groups, with significantly lower frequencies compared to both HR and MR (∗∗*p* = 0.009 and ∗*p* = 0.015, respectively). A trend toward higher frequencies of BA.2^+^ RBD MBC across the LR, MR, and HR was also observed, although not statistically significant (Figure 3H).

In summary, clustering of serological data identified three distinct groups of SOT recipients, each exhibiting a different capacity to develop SARS-CoV-2–specific antibody and MBC responses, highlighting the heterogeneity of vaccine-induced immune responses among SOT recipients.

### Identification of demographic and clinical factors associated with different spike-specific antibody responses

**Figure 4:**
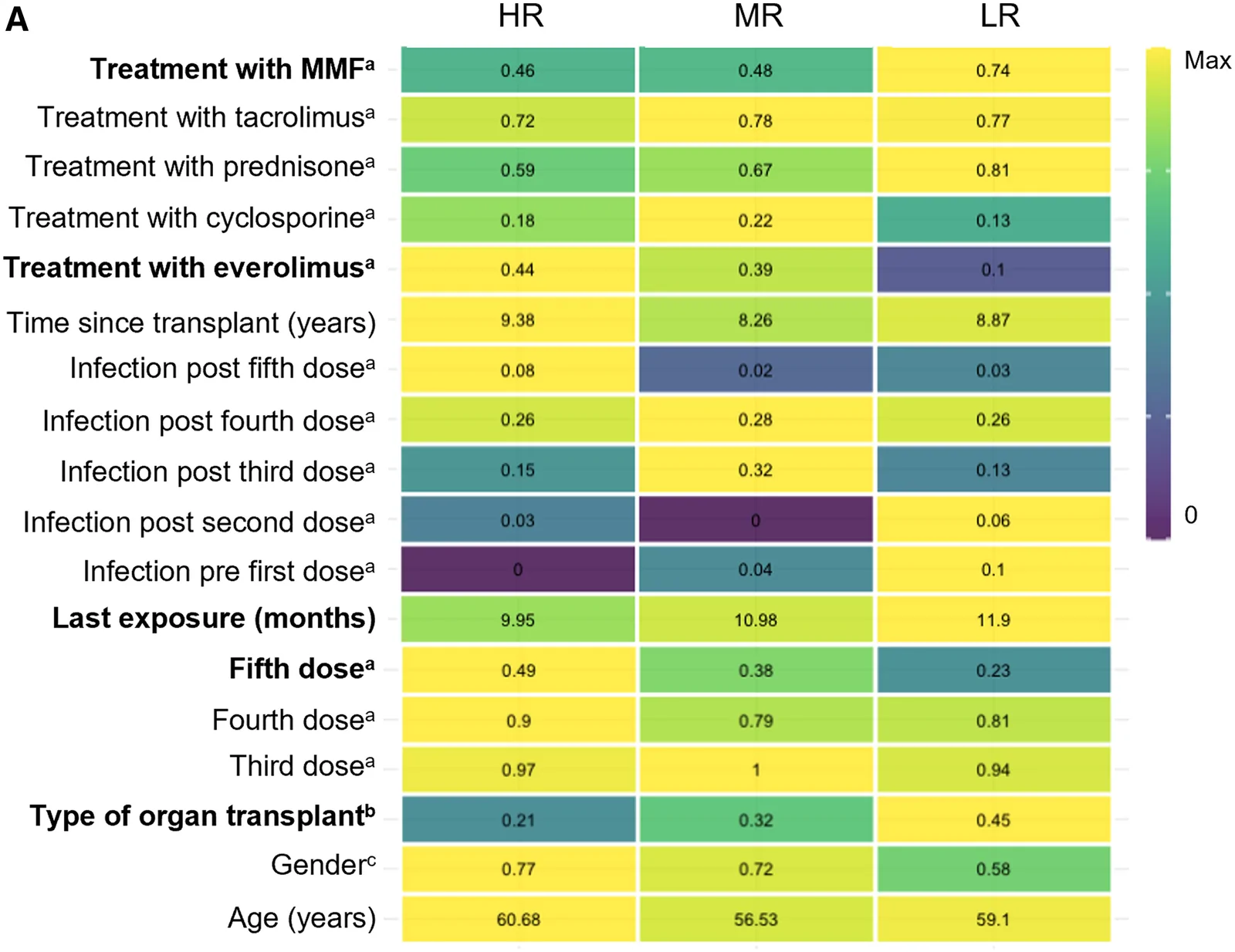
Univariable analysis of clinical factors among SOT recipient clusters of responders Clinical and demographic variables of each participant were collected and reported according to the categorization into clusters of responders. Cluster-specific means were scaled to [0,1] and depicted as a heatmap. Clinical variables with a significant impact on the classification within clusters of responders are reported in bold. Numbers within each box indicate the mean value of that variable in that cluster. Fisher’s exact test and Mann-Whitney test were used for comparing categorical and continuous variables between clusters. ^a^ For variables with a yes/no classification, the value 1 and 0 were assigned to individuals that have the feature (yes) and not (no), respectively; ^b^ for the “type of organ transplant” variable, the values 0 and 1 were assigned to the condition “heart transplant”, and “lung transplant”, respectively; ^c^ for the “sex” variable, the values 0 and 1 were assigned to the condition “female” and “male”, respectively. HR = high responders; MR = medium responders; LR = low responders; Last antigen exposure = time since the last vaccination or breakthrough infection.

Demographic and clinical parameters of SOT recipients were evaluated for their association with cluster categorization (Figure 4; Table 3). From the univariable comparisons (Fisher’s exact test), a significantly higher frequency of participants in the LR group received MMF treatment (70.97%) compared to those in the HR (46.15%, ∗*p* = 0.028) and MR groups (46.81%, ∗*p* = 0.020) (Figure 4; Table 3), indicating an association between MMF treatment and lower humoral responses against SARS-CoV-2. Notably, 48.72% of participants classified as HR received the fifth bivalent Omicron-adapted vaccine dose, versus 22.58% of LR participants (∗*p* = 0.028, Figure 4; Table 3), suggesting a positive association between fifth dose receipt and humoral vaccine responsiveness. Furthermore, HTx recipients predominated in the HR group (79.49%), while LuTx recipients were more frequently classified as LR (45.16%, ∗*p* = 0.038, Figure 4; Table 3), indicating differences in the distribution of clusters between transplant types. Participants clustered in LR had a significantly longer interval since their last vaccine administration or breakthrough infection compared to HR (median of 11 and 8 months, respectively, ∗*p* = 0.035, Figure 4; Table 3).

**Table 3:** Clinical variables among clusters of responders

Clinical variables	HR (*n* = 39)	MR (*n* = 47)	LR (*n* = 31)	Univariable statistical analyses – *p* value (ODDS ratio)		
				HR vs. MR	HR vs. LR	MR vs. LR
Treatment with MMF^a^ – n (%)	18 (46.15)	22 (46.81)	23 (74.19)	1 (0.974)	**0.028 (0.303)**	**0.020 (0.311)**
Treatment with tacrolimus^a^ – n (%)	28 (71.79)	37 (78.72)	24 (77.42)	0.615 (0.691)	0.784 (0.745)	1 (1.078)
Treatment with prednisone^a^ – n (%)	23 (58.97)	32 (68.08)	25(80.64)	0.499 (0.677)	0.071 (0.350)	0.299 (0.516)
Treatment with cyclosporine^a^ – n (%)	7 (17.95)	10 (21.28)	4 (12.90)	0.789 (0.811)	0.744 (1.468)	0.386 (1.811)
Treatment with everolimus^a^ – n (%)	17 (43.59)	18 (38.30)	3 (9.68)	0.664 (1.242)	**0.003 (7.017)**	**0.008 (5.675)**
Time since transplant (years) – median (IQR)	9 (3.5–14)	8 (3–11)	7 (4–13)	0.473	0.740	0.822
Infection post 5^th^ dose^a^ – n (%)	3 (7.69)	1 (2.13)	1 (3.23)	0.325 (0.261)	0.624 (0.400)	1 (1.533)
Infection post 4^th^ dose^a^ – n (%)	10 (25.64)	13 (27.66)	8 (25.81)	1 (0.903)	1 (0.991)	1 (1.098)
Infection post 3^rd^ dose^a^ – n (%)	6 (15.38)	15 (31.91)	4 (12.90)	0.085 (0.392)	1 (1.224)	0.064 (3.120)
Infection post 2^nd^ dose^a^ – n (%)	1 (2.56)	0 (0)	2 (6.45)	0.453 (0)	0.580 (2.620)	0.155 (inf)
Infection pre 1^st^ dose^a^ – n (%)	0 (0)	2 (2.13)	3 (9.68)	0.498 (inf)	0.082 (inf)	0.381 (2.411)
Last antigen exposure (months) – median (IQR)	8 (7–12)	10 (7.5–14)	11 (8–15)	0.250	**0.036**	0.257
5^th^ dose^a^ – n (%)	19 (48.72)	18 (38.30)	7 (22.58)	0.385 (1.523)	**0.028 (3.201)**	0.215 (2.108)
4^th^ dose^a^ – n (%)	35 (89.74)	37 (78.72)	25 (80.64)	0.242 (2.342)	0.320 (2.077)	1 (0.889)
3^rd^ dose^a^ – n (%)	38 (97.44)	47 (100)	29 (93.55)	0.453 (inf)	0.580 (0.381)	0.155 (inf)
Type of organ transplant^b^ – n (%)	/	/	/	0.328 (0.554)	**0.038 (0.319)**	0.338 (0.573)
Heart transplant	31 (79.49)	32 (68.09)	17 (54.84)	/	/	/
Lung transplant	8 (20.51)	15 (31.91)	14 (45.16)	/	/	/
Sex^c^ – n (%)	/	/	/	0.804 (1.271)	0.122 (2.376)	0.225 (1.873)
Female	9 (23.08)	13 (27.66)	13 (41.94)	/	/	/
Male	30 (76.92)	34 (72.34)	18 (58.06)	/	/	/
Age – median (IQR)	61 (56–68)	57 (50–65)	61 (53–66)	0.099	0.723	0.342

Mann-Whitney and Fisher’s exact tests were applied to perform univariable comparisons of continuous and categorical variables across responder groups, respectively. ∗*p* < 0.05 and corresponding Odds ratio are highlighted in bold. OR = Odds Ratio; HR = high responders; MR = medium responders; LR = low responders.

a For variables with a yes/no condition, the values 0 and 1 were assigned to individuals who have the condition “no” and “yes”, respectively.

b For the “type of organ transplant” variable, the values 0 and 1 were assigned to the conditions “heart transplant” and “lung transplant”, respectively.

c For the “sex” variable, the values 0 and 1 were assigned to the condition “female” and “male”, respectively.

A LASSO regression model was fitted to complement the univariable analyses and investigate the independent association of demographic and clinical parameters with humoral vaccine responsiveness in a multivariate framework (Table S3). Model significance was assessed using a permutation-based approach, yielding an overall ∗*p* of 0.014. Variables with non-zero coefficients were selected by the model as relevant descriptors independently associated with differences in humoral vaccine responsiveness. For each cluster of responders, a positive coefficient indicated that the corresponding variable increased the likelihood of being classified in that cluster, whereas a negative coefficient indicated a lower likelihood. Among the variables selected by the model as relevant for the LR classification, treatment with MMF and lung transplantation were associated with a higher likelihood of being classified as low responder (coefficients of 0.07 and 0.08, respectively, Table S3). On the contrary, treatment with everolimus, administration of the fifth dose, and male sex were associated with a lower likelihood of being classified as low responder (coefficients of −0.79, −0.27, −0.06, respectively). Additional variables identified as relevant by the LASSO model included infection after the 3^rd^ dose and age (coefficients of 0.50 and −0.01, respectively) for the MR classification, and time since the last antigen exposure and transplanted organ type (coefficients of −0.01 and −0.14, respectively) for the HR classification.

A consensus approach was applied, retaining only variables consistently identified in both the univariable analysis and the LASSO regression model. Both univariable analysis and the LASSO model identified the transplanted organ type, the administration of the fifth bivalent Omicron-adapted dose, and immunosuppressive pharmacological treatments (everolimus and MMF) as variables associated with different vaccine responsiveness. The association of all these variables with the magnitude and quality of RBD-specific MBC response was then assessed.

### RBD-specific memory B cell response differs between heart and lung transplant recipients

**Figure 5:**
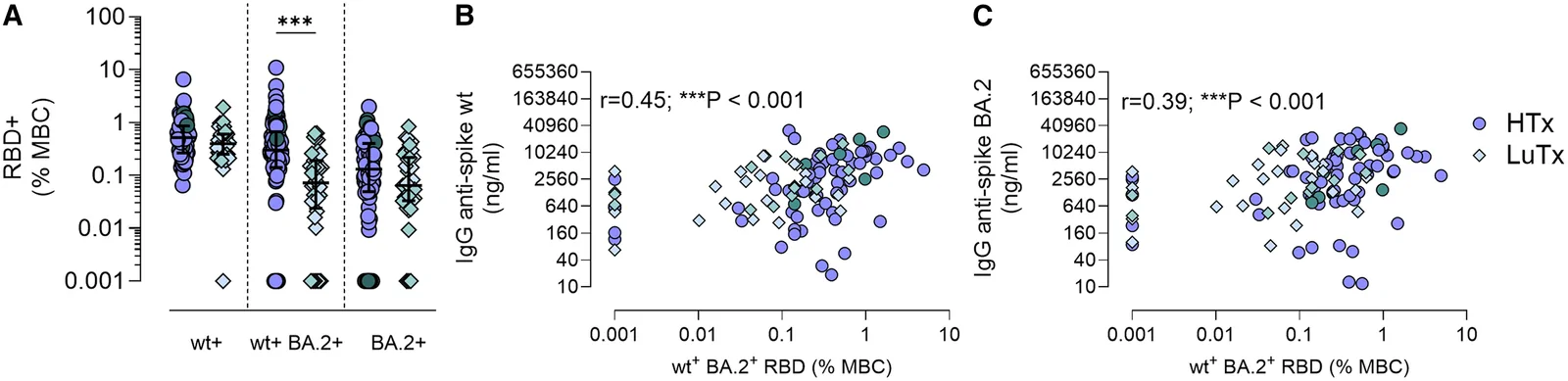
RBD-specific MBC response in HTx and LuTx recipients (A) Frequency of wt^+^ RBD, wt^+^BA.2^+^ and BA.2^+^ RBD MBC assessed in the groups of heart transplant recipients (HTx recipients, circles) and lung transplant recipients (LuTx recipients, diamond). (B and C) Spearman correlation between the wt^+^BA.2^+^ RBD MBC frequencies and the wt- (B) or BA.2 spike-specific IgG concentrations (C) across the entire SOT recipients cohort. Data are shown as dot plot, with the median and error bars representing the interquartile range. Mann-Whitney test was used for assessing statistical differences between HTx and LuTx recipient groups in (A). HTx and LuTx recipients who had received SARS-CoV-2 vaccination prior to organ transplantation are highlighted in dark green and light green, respectively. ∗∗∗*p* < 0.001. Sample size: HTx recipient group (A–C: *n* = 73); LuTx recipient group (A–C: *n* = 36).

Considering that HTx recipients were mostly distributed among HR and LuTx recipients among LR, the RBD-specific MBC responses was analyzed according to the transplanted organ type. While the two groups showed comparable frequencies of wt^+^ and BA.2^+^ RBD MBC, the HTx recipient group showed a significantly higher frequency of wt^+^BA.2^+^ RBD MBC compared to the LuTx recipient group (∗∗∗*p* < 0.001, U Mann-Whitney value = 615, Figure 5A).

To determine whether this enrichment correlated with the humoral response, Spearman correlation analysis was performed between wt^+^BA.2^+^ RBD MBC frequencies and wt- and BA.2 spike-specific IgG levels across all SOT recipients. wt^+^BA.2^+^ RBD MBC positively correlated with both wt- and BA.2 spike-specific IgG concentrations (*r* = 0.45 and *r* = 0.39, respectively, both ∗∗∗*p* < 0.001, Figures 5B and 5C), suggesting that the expansion of wt^+^BA.2^+^ RBD MBC is functionally linked to the humoral vaccine response.

### Association of the fifth bivalent Omicron-adapted vaccine dose with RBD-specific memory B cell phenotypes

**Figure 6:**
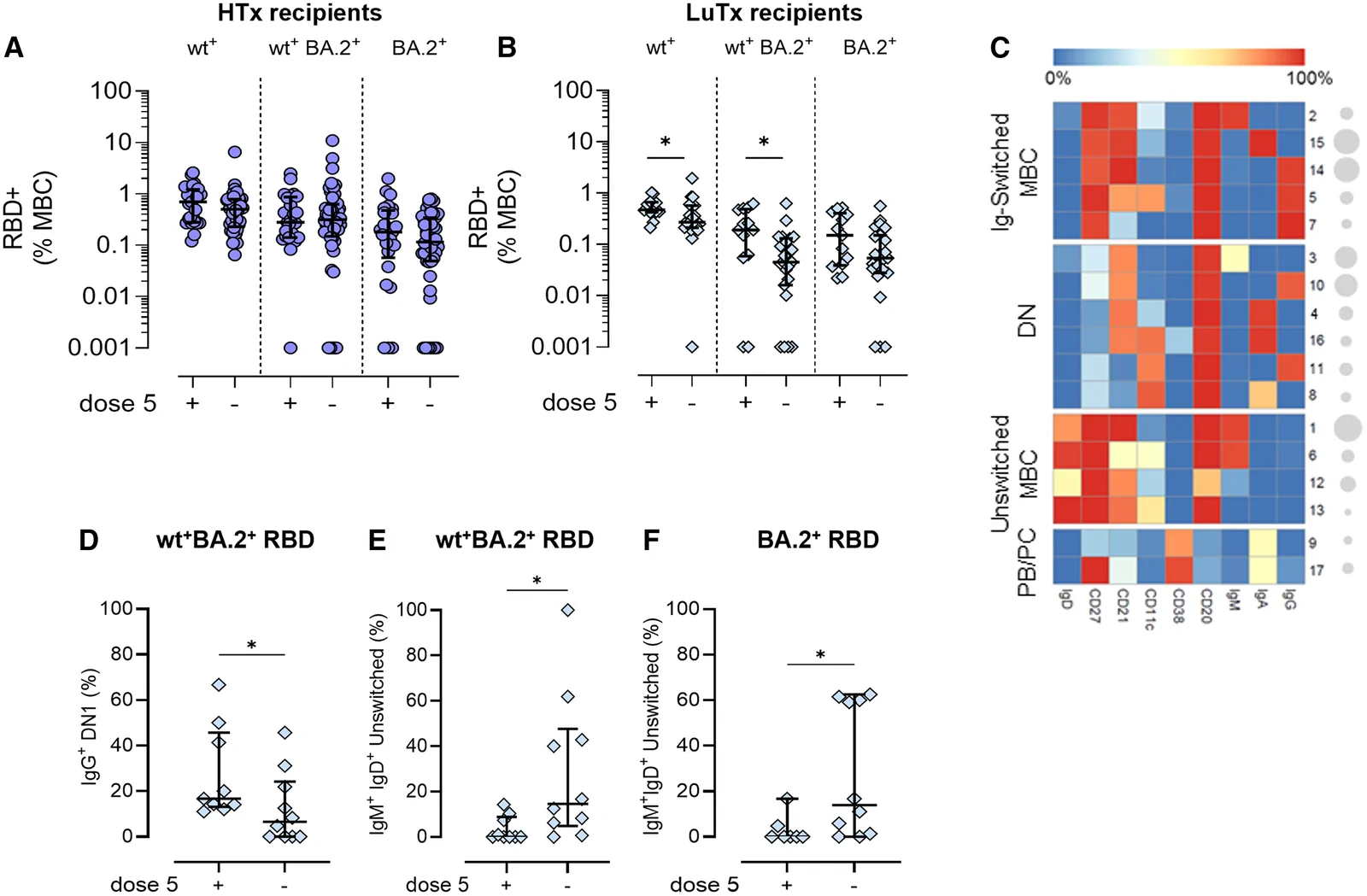
wt^+^ RBD, wt^+^BA.2^+^ and BA.2^+^ RBD MBC subsets among HTx and LuTx recipient groups with or without the fifth bivalent Omicron-adapted vaccine dose (A and B) Frequency of wt^+^, wt^+^BA.2^+^ and BA.2^+^ RBD MBC assessed in the groups of heart transplant recipients (HTx, A) and lung transplant recipients (LuTx, B) who received and did not receive the fifth bivalent Omicron-adapted vaccine dose. (C) Heatmap of clusters obtained from the FlowSOM analysis of total CD19^+^ MBC. Markers (IgD, CD27, CD21, CD11c, CD38, CD20, IgM, IgA, IgG) are reported in column, and the identified clusters in row. Clusters are grouped in Ig-switched MBC, double-negative (DN), unswitched MBC and plasmablasts/plasmacells (PB/PC). The percentage of cells positive for each marker is visualized with a color scale from blue (0%) to red (100%). (D–F) Frequency of IgG^+^ DN1 wt^+^BA.2^+^ RBD MBC (D), IgM^+^ IgD^+^ unswitched wt^+^BA.2^+^ (E) and IgM^+^ IgD^+^ unswitched BA.2^+^ RBD MBC (H), in LuTx recipients who received or not the fifth dose. Data in A) – B) and D) – F) are shown as dot plot, with the median and error bars representing the interquartile range. Mann-Whitney test was used for assessing statistical differences between participants who received the fifth dose and those who did not in A, B and D–F. ∗*p* < 0.05. Sample size: HTx recipient group who received the fifth dose (dose 5 +, A: *n* = 25); HTx recipient group who did not receive the fifth dose (dose 5 -, A: *n* = 48); LuTx recipient group who received the fifth dose (dose 5 +, B: *n* = 13; D-E: *n* = 9; F: *n* = 7); LuTx recipient group who did not receive the fifth dose (dose 5 -, B: *n* = 23; D-F: *n* = 10).

Given the association between the administration of the bivalent Omicron-adapted vaccine (fifth dose) and the higher antibody responses, the frequencies of wt^+^ RBD, wt^+^BA.2^+^ RBD and BA.2^+^ RBD MBC were assessed within the HTx and LuTx recipient groups, stratified between those who received or did not receive the fifth vaccine dose. HTx recipients showed similar frequencies of wt^+^ RBD, wt^+^BA.2^+^ RBD and BA.2^+^ RBD MBC, regardless of fifth dose administration (Figure 6A). In contrast, LuTx recipients who received the fifth dose showed significantly higher frequencies of both wt^+^ RBD and wt^+^BA.2^+^ RBD than those who did not receive it (∗*p* = 0.030 and ∗*p* = 0.016, U Mann-Whitney values = 84 and 77, respectively, Figure 6B).

To deeply characterize the MBC response among SOT recipients, phenotypes of wt^+^ RBD, wt^+^BA.2^+^ RBD and BA.2^+^ RBD MBC were profiled within the HTx and LuTx recipients who received or did not receive the fifth dose. A FlowSOM analysis was performed on the total MBC compartment based on the surface expression of CD27, CD21, CD11c, CD38, CD20, IgD, IgM, IgA, and IgG markers. The analysis identified a total of 17 MBC clusters (Figure 6C), that were grouped into Ig-switched MBC (IgD^−^ CD27^+^), double-negative (DN; IgD^−^ CD27^−^), unswitched MBC (IgD^+^ CD27^+^) and plasmablast/plasma cells (PB/PC; CD20^−^ CD38^+^). The phenotypic profile of RBD-specific MBC was then determined by assessing the frequency of each cluster within wt^+^ RBD, wt^+^BA.2^+^ RBD and BA.2^+^ RBD MBC. Four populations comprised the majority of the wt^+^ RBD, wt^+^BA.2^+^ RBD and BA.2^+^ RBD specific MBC, such as IgG^+^ and IgM^+^IgD^+^ resting memory cells (cluster 14 and 1, respectively), IgG^+^ DN1 B cells (IgD^−^ CD27^−^ CD21^+^ CD11c^−^, cluster 10) and IgG^+^ DN2 B cells (IgD^−^ CD27^−^ CD21^−^ CD11c^+^, cluster 11, Table S4). The distribution of RBD-specific MBC among these predominant populations was then analyzed within HTx and LuTx recipient groups who received or did not receive the fifth bivalent Omicron-adapted dose (Figures 6D–6F; Table S5). Within the HTx recipient group, administration of the fifth vaccine dose was not associated with significant differences in the 4 most represented subsets across wt^+^ RBD, wt^+^BA.2^+^ RBD and BA.2^+^ RBD MBC. On the contrary, the administration of the fifth dose within the LuTx recipient group was associated with major differences in the phenotypic profile of antigen-specific MBC. In particular, LuTx recipients who received the fifth dose displayed significantly higher frequencies of IgG^+^ DN1 wt^+^BA.2^+^ MBC (∗*p* = 0.049, U Mann-Whitney value = 21, Figure 6D), as well as significantly lower frequencies of IgM^+^IgD^+^ wt^+^BA.2^+^ and BA.2^+^ RBD MBC (∗*p* = 0.017 and ∗*p* = 0.030, U Mann-Whitney values = 16.50 and 13.50, Figures 6E and 6F, respectively) compared to LuTx recipients who did not receive the fifth dose.

In summary, vaccination with a fifth bivalent Omicron-adapted vaccine dose was associated with distinct phenotypic profiles of RBD-specific MBCs in LuTx recipients. The expansion of IgG^+^ DN1 wt^+^BA.2^+^ RBD MBC and the reduction of IgM^+^IgD^+^ unswitched wt^+^BA.2^+^ and BA.2^+^ RBD MBC suggest a phenotypic profile consistent with a more differentiated and class-switched B cell response.

### Spike-specific humoral and memory B cell responses according to immunosuppressive treatments

The third factor identified by the clustering analysis that was associated with a lower antibody response was the immunosuppressive treatment. Indeed, individuals classified as lower responders were more often treated with MMF, and less frequently received everolimus. Therefore, the association of different immunosuppressive treatments with the RBD-specific MBC response was investigated among HTx and LuTx recipient groups, separately.

**Figure 7:**
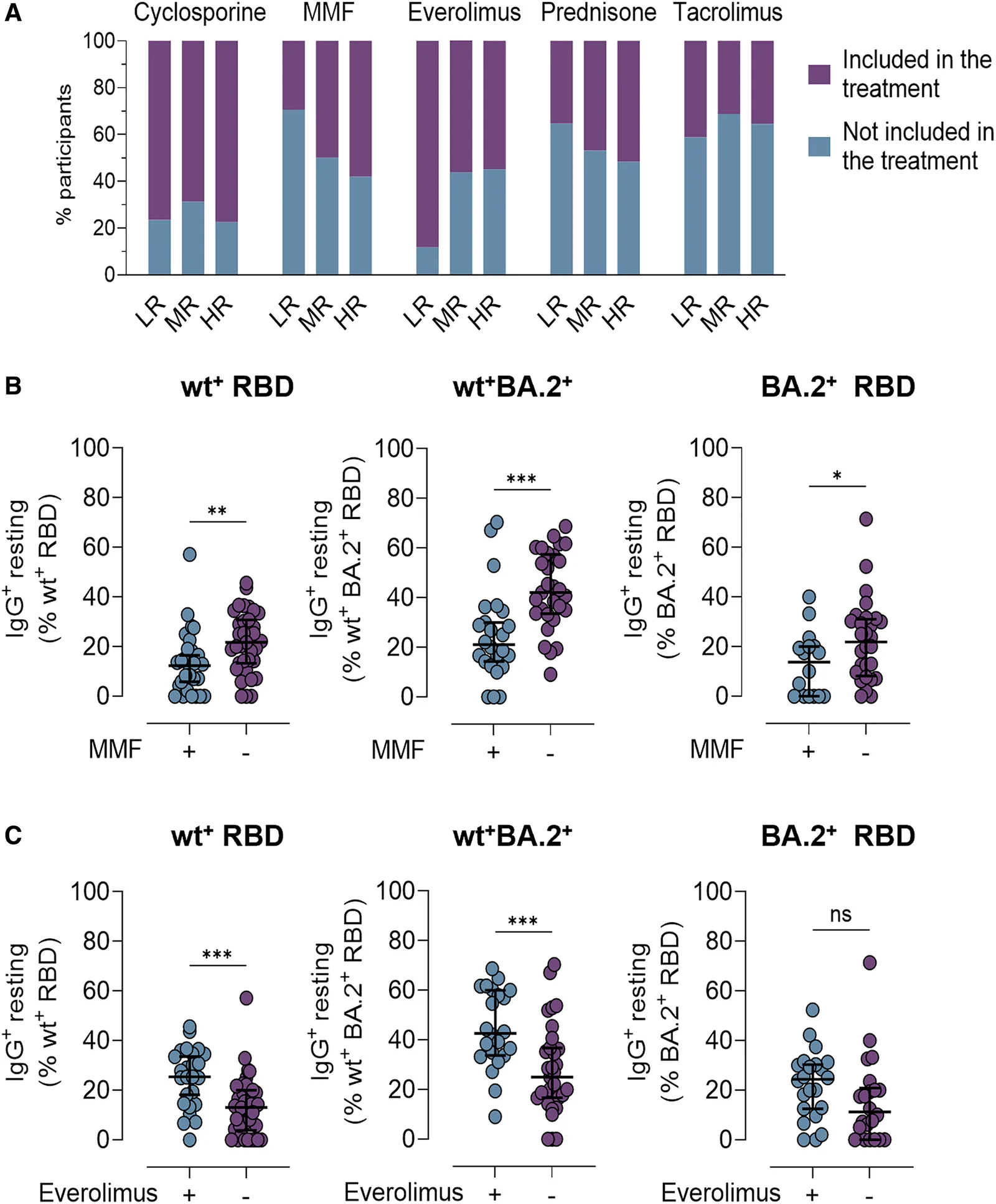
Impact of immunosuppressive treatments on B cell responses among HTx recipient group (A) Immunosuppressive treatment data were summarized by proportional counts and rendered as stacked barplots according to the stratification into the high responders (HR), medium responders (MR) and low responders (LR) clusters. (B) Frequencies of IgG resting wt^+^ (left image), wt^+^BA.2^+^ (middle image) and BA.2^+^ (right image) RBD MBC in HTx recipient group treated or not with MMF. (C) Frequencies of IgG resting wt^+^ (left image), wt^+^BA.2^+^ (middle image) and BA.2^+^ (right image) RBD MBC in HTx recipient group treated or not with everolimus. Data are shown as dot plot, with the median and error bars representing the interquartile range. Mann-Whitney test was used for assessing statistical differences between groups in (B and C). ∗*p* < 0.05, ∗∗*p* < 0.01, ∗∗∗*p* < 0.001. Sample size: MMF + HTx (B, left image: *n* = 28; B, middle image: *n* = 24; B, right image: *n* = 16); MMF- HTx (B, left image: *n* = 35; B, middle image: *n* = 29; B, right image: *n* = 28). Everolimus+ HTx (C, left image: *n* = 25; C, middle image: *n* = 22; C, right image: *n* = 22); everolimus- HTx (C, left image: *n* = 38; C, middle image: *n* = 32; C, right image: *n* = 22). MMF = mycophenolate mofetil.

As reported in Table 2, HTx recipients were treated with one (low regimen), two (medium regimen), or three (high regimen) drugs, which included different combination of calcineurin inhibitors (cyclosporine and tacrolimus), corticosteroids (prednisone), mycophenolate mofetil (MMF), and mammalian target of rapamycin inhibitor (mTORi, Everolimus). The distribution of HTx recipients undergoing different immunosuppressive treatments among HR, MR, and LR groups is shown in Figure 7A. Consistent with the results of the univariable and LASSO analyses, HTx recipients treated with everolimus were underrepresented in the LR group, while those receiving MMF showed the opposite trend. Given these findings, the association of MMF and everolimus with the RBD-specific MBC response was investigated by analyzing the frequency of the IgG^+^ resting subset, that represented the predominant IgG^+^ resting MBC population identified by the computational analysis in HTx recipients treated or not with each of these drugs (Figures 7B and 7C). MMF treatment was associated with significantly lower frequencies of IgG^+^ resting MBC across wt^+^, wt^+^BA.2^+^ and BA.2^+^ RBD MBC compared to MMF untreated HTx recipients (Figure 7B, ∗∗*p* = 0.004, ∗∗∗*p* < 0.001 and ∗*p* = 0.039, U Mann-Whitney values = 284, 144.5 and 140, respectively), suggesting a negative role of this pharmacological treatment on the vaccine-induced immune response. Conversely, treatment with everolimus showed an opposite trend, being associated with significantly higher frequencies of IgG^+^ resting MBC across wt^+^ and wt^+^BA.2^+^ RBD MBC (Figure 7C, left and middle images, both ∗∗∗*p* < 0.001, U Mann-Whitney values = 213 and 161, respectively). HTx treated with everolimus also displayed a trend toward higher frequencies of IgG^+^ resting BA.2^+^ RBD MBC compared to the untreated ones, although not statistically significant (*p* = 0.055).

**Figure 8:**
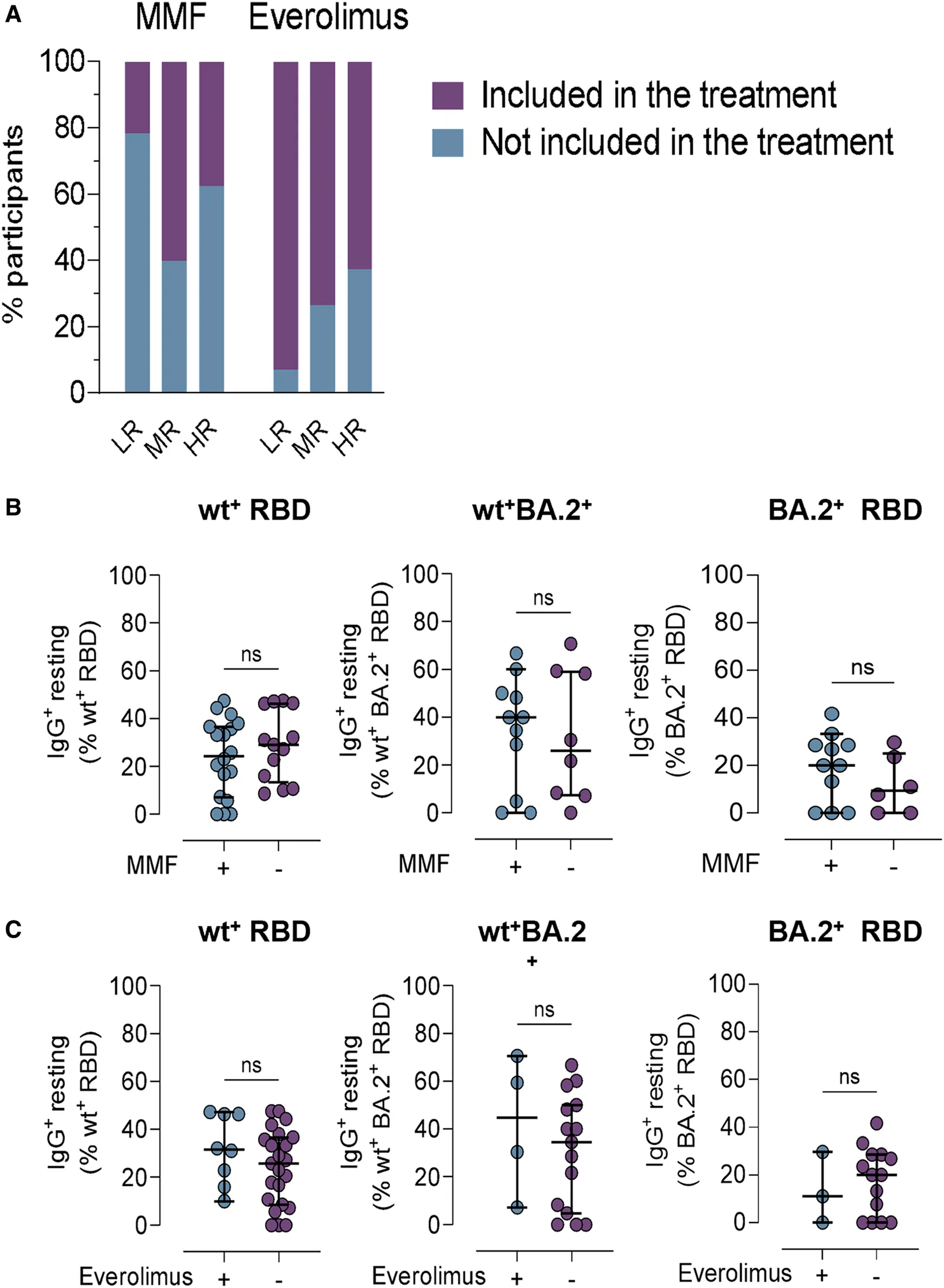
Impact of immunosuppressive treatments on B cell responses among LuTx recipients (A) Treatment with everolimus or MMF were summarized by proportional counts and rendered as stacked bar plots according to the stratification into the high responders (HR), medium responders (MR), and low responders (LR) clusters. (B) Frequencies of IgG resting wt^+^ (left image), wt^+^BA.2^+^ (middle image) and BA.2^+^ (right image) RBD MBC in LuTx recipient group treated or not with MMF. (C) Frequencies of IgG resting wt^+^ (left image), wt^+^BA.2^+^ (middle image) and BA.2^+^ (right image) RBD MBC in LuTx recipient group treated or not with everolimus. Data are shown as dot plot, with the median and error bars representing the interquartile range. Mann-Whitney test was used for assessing statistical differences between groups in (B and C). Sample size: MMF + LuTx (B, left image: *n* = 18; B, middle image: *n* = 11; B, right image: *n* = 11); MMF- LuTx (B, left image: *n* = 13; B, middle image: *n* = 8; B, right image: *n* = 6). Everolimus+ LuTx (C, left image: *n* = 8; C, middle image: *n* = 4; C, right image: *n* = 3); everolimus- LuTx (C, left image: *n* = 23; C, middle image: *n* = 15; C, right image: *n* = 14). MMF = mycophenolate mofetil.

Within the LuTx recipient group, immunosuppressive regimens (Table 2) consistently included a combination of corticosteroids (Prednisone) and calcineurin inhibitors (Tacrolimus) (P + T; Group I). In some participants, these were supplemented with MMF (P + T + MMF; Group II) or mTOR inhibitors (Everolimus) (P + T + E; Group III). The inclusion of everolimus or MMF among LuTx recipients classified as HR, MR, or LR is shown in Figure 8A. The association of MMF and everolimus treatment with the RBD-specific MBC response was then assessed by analyzing the frequency of the IgG^+^ resting subset within wt^+^, wt^+^BA.2^+^ and BA.2^+^ RBD MBC in recipients treated or not with each of these agents. No significant differences in the frequencies of wt^+^, wt^+^BA.2^+^ and BA.2^+^ RBD IgG^+^ resting MBC were observed among LuTx recipients receiving or not receiving everolimus and MMF (Figures 8B and 8C).

In summary, MMF and everolimus treatments were associated with distinct humoral and RBD-specific MBC responses in the HTx recipient group, while no significant differences were observed in the LuTx recipients undergoing different immunosuppressive regimens.

### Multivariable identification of factors associated with distinct spike-specific memory B cell responses

Generalized linear models (GLM) with binomial distribution were fitted to investigate the independent association of demographic and clinical variables, including treatment with immunosuppressive drugs, receipt of the fifth bivalent vaccine dose, age, and sex, with the total frequencies of wt^+^, wt^+^BA.2^+^ and BA.2^+^ RBD MBC, as well as the differential abundance of the 4 major RBD-specific MBC phenotypes (IgG^+^ resting, IgM^+^IgD^+^ resting, IgG^+^ DN1 and IgG^+^ DN2 B cells; Table 4).

**Table 4:** Multivariable analysis of factors impacting on the spike-specific MBC response

	Clinical variables – estimate (*p* value)							
	Age	Sex^a^	5^th^ dose	Treatment with everolimus^b^	Treatment with cyclosporine^b^	Treatment with prednisone^b^	Treatment with tacrolimus^b^	Treatment with MMF^b^
**HTx recipients**								

wt^+^ RBD MBC^c^	0.02 (0.630)	0.74 (0.329)	1.01 (0.145)	−0.28 (0.758)	−17.29 (0.992)	−0.57 (0.355)	−17.49 (0.992)	−1.29 (0.141)
wt^+^ RBD MBC - IgD^+^IgM^+^ resting^d^	−0.04 (0.293)	−1.16 (0.160)	0.53 (0.471)	−1.16 (0.229)	16.48 (0.993)	−0.09 (0.89)	16.54 (0.993)	0.69 (0.441)
wt^+^ RBD MBC - IgG^+^ DN1^d^	0.06 (0.064)	−0.57 (0.438)	−0.20 (0.762)	1.61 (0.094)	−0.01 (0.992)	0.59 (0.333)	0.28 (0.834)	0.19 (0.828)
wt^+^ RBD MBC - IgG^+^ resting^d^	−0.01 (0.733)	−1.16 (0.121)	−0.21 (0.772)	1.24 (0.19)	−16.53 (0.993)	0.17 (0.782)	−16.87 (0.993)	−0.61 (0.474)
wt^+^ RBD MBC - IgG^+^ DN2^d^	/	/	/	/	/	/	/	/
wt^+^ BA.2^+^ RBD MBC^c^	0.07 (0.137)	−0.31 (0.756)	1.1 (0.226)	−1.75 (0.169)	−16.43 (0.992)	−0.98 (0.204)	−18 (0.991)	**−3.32 (0.011)**
wt^+^ BA.2^+^ RBD MBC - IgD^+^IgM^+^ resting^d^	/	/	/	/	/	/	/	/
wt^+^ BA.2^+^ RBD MBC - IgG^+^ DN1^d^	−0.03 (0.427)	−0.77 (0.343)	−0.01 (0.988)	**3.13 (0.021)**	0.63 (0.602)	−0.23 (0.715)	0.86 (0.464)	2.00 (0.117)
wt^+^ BA.2^+^ RBD MBC - IgG^+^ resting^d^	0.07 (0.136)	−0.95 (0.309)	−0.89 (0.305)	0.50 (0.666)	−0.27 (0.857)	0.05 (0.942)	−0.16 (0.912)	**−2.26 (0.035)**
wt^+^ BA.2^+^ RBD MBC - IgG^+^ DN2^d^	/	/	/	/	/	/	/	/
BA.2^+^ RBD MBC^c^	0.13 (0.018)	−0.47 (0.650)	−0.02 (0.982)	1.32 (0.242)	−16.87 (0.993)	−0.52 (0.499)	−16.63 (0.993)	0.86 (0.439)
BA.2^+^ RBD MBC - IgD^+^IgM^+^ resting^d^	/	/	/	/	/	/	/	/
BA.2^+^ RBD MBC - IgG^+^ DN1^d^	−0.05 (0.403)	2.82 (0.051)	**−2.67 (0.035)**	1.35 (0.271)	−2.97 (0.103)	−1.23 (0.158)	−0.44 (0.778)	0.21 (0.859)
BA.2^+^ RBD MBC - IgG^+^ resting^d^	−0.05 (0.299)	1.80 (0.108)	−1.28 (0.129)	0.88 (0.361)	−1.89 (0.229)	0.19 (0.79)	−0.99 (0.482)	0.34 (0.725)
BA.2^+^ RBD MBC - IgG^+^ DN2^d^	**0.16 (0.038)**	−2.14 (0.116)	**2.23 (0.046)**	−2.05 (0.081)	19.84 (0.991)	0.13 (0.877)	17.97 (0.992)	−1.55 (0.19)

**LuTx recipients**								

wt^+^ RBD MBC^c^	0.03 (0.428)	−0.54 (0.497)	1.36 (0.101)	−1.48 (0.273)	/	/	/	−0.35 (0.751)
wt^+^ RBD MBC - IgD^+^IgM^+^ resting^d^	/	/	/	/	/	/	/	/
wt^+^ RBD MBC - IgG^+^ DN1^d^	−3.34 (0.401)	0.04 (0.13)	−1.32 (0.36)	0.76 (0.374)	/	/	/	1.25 (0.126)
wt^+^ RBD MBC - IgG^+^ resting^d^	4.34 (0.062)	−0.09 (0.588)	0.46 (0.157)	−1.27 (0.182)	/	/	/	1.95 (0.893)
wt^+^ RBD MBC - IgG^+^ DN2^d^	/	/	/	/	/	/	/	/
wt^+^ BA.2^+^ RBD MBC^c^	/e	/e	/e	/e	/e	/e	/e	/e
wt^+^ BA.2^+^ RBD MBC - IgD^+^IgM^+^ resting^d^	−0.50 (0.633)	0.023 (0.464)	0.84 (0.082)	−2.68 (0.28)	/	/	/	−2.06 (0.516)
wt^+^ BA.2^+^ RBD MBC - IgG^+^ DN1^d^	−3.16 (0.585)	0.03 (0.302)	−1.22 (0.26)	1.37 (0.453)	/	/	/	1.35 (0.236)
wt^+^ BA.2^+^ RBD MBC - IgG^+^ resting^d^	4.43 (0.073)	−0.12 (0.952)	0.07 (0.712)	0.52 (0.261)	/	/	/	2.16 (0.272)
wt^+^ BA.2^+^ RBD MBC - IgG^+^ DN2^d^	/	/	/	/	/	/	/	/
BA.2^+^ RBD MBC^d^	0.04 (0.537)	−2.35 (0.116)	1.92 (0.201)	−3.15 (0.248)	/	/	/	−1.20 (0.571)
BA.2^+^ RBD MBC - IgD^+^IgM^+^ resting^d^	22.94 (0.308)	−0.08 (0.253)	−1.90 (0.454)	−1.15 (0.997)	/	/	/	−17.40 (0.997)
BA.2^+^ RBD MBC - IgG^+^ DN1^d^	−21.89 (0.191)	0.09 (0.464)	1.04 (0.866)	0.22 (0.995)	/	/	/	16.25 (0.995)
BA.2^+^ RBD MBC - IgG^+^ resting^d^	−3.34 (0.343)	0.06 (0.223)	1.57 (0.875)	0.19 (0.744)	/	/	/	−0.68 (0.513)
BA.2^+^ RBD MBC - IgG^+^ DN2^d^	/	/	/	/	/	/	/	/

Generalized linear models (GLM) were fitted to evaluate the independent association of immunosuppressive treatments with the abundance of RBD-specific MBC phenotypes, adjusting for fifth dose administration, age, and sex. Estimates and corresponding ∗*p* < 0.05 are highlighted in bold.

a For the “sex” variable the value 0 and 1 were assigned to the condition “female” and “male”, respectively.

b For variables with a yes/no condition, the value 0 and 1 were assigned to individuals that have the condition “no” and “yes”, respectively.

c Frequencies were calculated relative to total memory B cells.

d Frequencies were calculated relative to the relative RBD-specific MBC population.

e The model did not converge due to small sample size.

From the multivariable analysis, administration of the fifth bivalent Omicron-adapted vaccine dose was associated to phenotypic changes within the HTx recipient group. Particularly, administration of the fifth dose was inversely associated with IgG^+^ DN1 BA.2^+^ RBD MBC frequencies (estimate = −2.67, ∗*p* = 0.035) and positively associated with IgG^+^ DN2 BA.2^+^ RBD MBC frequencies (estimate = 2.23, ∗*p* = 0.046). Within HTx recipients, treatment with everolimus was significantly associated with higher frequencies of IgG^+^ DN1 wt^+^BA.2^+^ RBD MBC (estimate = 3.13, ∗*p* = 0.021), while MMF treatment was inversely associated with both the total frequencies of wt^+^BA.2^+^ RBD MBC (estimate = −3.32, ∗*p* = 0.011) and the frequencies of IgG^+^ wt^+^BA.2^+^ RBD MBC (estimate = −2.26, ∗*p* = 0.035). Older age was also positively associated with higher frequencies of IgG^+^ DN2 BA.2^+^ RBD MBC (estimate = 0.157, ∗*p* = 0.038). In contrast to HTx recipients, no significant associations were observed in LuTx recipients, possibly due to the smaller sample size and collinearity among covariates, which may have limited the ability to resolve the independent contribution of individual factors when modeled simultaneously. A positive association between the fifth dose and the frequencies of wt^+^BA.2^+^ RBD MBC was observed, with 88% of LuTx recipients who received the fifth dose and the upper 50% of wt^+^BA.2^+^ RBD MBC frequencies, while 88% of those who did not fell in the lower 50%. However, the model did not converge due to quasi-complete separation, likely reflecting the small sample size.

## Discussion

In this study, we applied computational models to identify key factors associated with the spike-specific humoral and memory B cell responses 2 years after the first SARS-CoV-2 vaccine in a group of HTx and LuTx recipients.

In the context of vaccinology, computational approaches such as dimensionality reduction and clustering algorithms enable the analysis of multidimensional serological datasets that integrate immunological, clinical, and pharmacological parameters, allowing the reduction of system complexity and the identification of key factors influencing vaccine responsiveness in specific patient populations, as exemplified here in heart and lung transplant recipients. By applying dimensionality reduction and clustering algorithms, we demonstrated that the type of organ transplanted, the administration of the fifth bivalent, Omicron-adapted dose, and the immunosuppressive pharmacological treatment were variables associated with differences in spike-specific humoral and MBC responses.

Computational analysis of four serological datasets, including IgG concentrations and ACE2/RBD binding inhibition capacity against wild-type and Omicron BA.2, clustered SOT recipient group into high, medium, and low responders. LuTx recipients were preferentially classified as low rather than high responders. This finding is consistent with previous studies, which highlighted the poor SARS-CoV-2 humoral response in LuTx recipients compared to other classes of SOT recipients at an early time point after vaccination.^23^^,^^24^ In addition, our data demonstrated that LuTx recipients developed an RBD-specific MBC response, although at significantly lower frequency than HTx recipients. The impaired immune response to SARS-CoV-2 vaccination observed in the LuTx recipients’ group may be due to an altered baseline immune state that has been previously linked to poor SARS-CoV-2 vaccine response in this population.^25^ Additionally, the reduced immunogenicity of vaccines observed in our group of LuTx recipients could also be due to the shorter time interval from transplantation to vaccination, a factor which is known to contribute to vaccine failure.^26^^,^^27^

From the clustering analysis, the administration of the fifth bivalent Omicron-adapted vaccine dose was associated with higher humoral responses, consistent with previous studies.^28^^,^^29^ When investigating the RBD-specific MBC response, while HTx recipients showed comparable frequencies of RBD-specific MBC irrespective of the administration of the fifth dose, LuTx recipients who received the fifth dose showed significantly higher frequencies of wt^+^ and wt^+^BA.2^+^ RBD MBC, suggesting an association between additional antigen exposure and higher MBC frequencies in this group. The RBD-specific MBC phenotypes were also profiled between HTx and LuTx recipients who did or did not receive the fifth bivalent Omicron-adapted vaccine dose. Both HTx and LuTx recipients exhibited comparable phenotypic profiles among the wt^+^ RBD MBC, irrespective of the administration of the fifth dose, possibly consistent with a well-established wt^+^ RBD MBC response, shaped by repeated exposure to the wt RBD from previous vaccination/infection.

On the contrary, phenotypic changes were observed among both wt^+^BA.2^+^ and BA.2^+^RBD MBC responses, with distinct patterns observed between the two transplant groups. Among the LuTx recipients group, administration of the fifth dose was associated with higher frequencies of IgG^+^ DN1 wt^+^BA.2^+^ RBD MBC and with lower frequencies of IgM^+^IgD^+^ resting wt^+^BA.2^+^ RBD and BA.2^+^ RBD MBC. The origin of IgG^+^ DN1 B cells is thought to be GC-dependent, either resulting from premature GC reaction exit or deriving from MBC that have lost CD27 expression following chronic antigenic stimulation.^30^ The expansion of this subset alongside the contraction of unswitched MBC is consistent with a shift toward more class-switched and potentially more affine MBC responses. This contrasts with observations in healthy individuals, who are influenced by immune imprinting,^31^^,^^32^^,^^33^^,^^34^ and suggests that SOT recipients, due to their weaker initial response to vaccination, may retain the ability to generate new clonal responses to viral variants, possibly because of a reduced recall of pre-existing clones (Costagli et al., in preparation). Given these data, boosting with vaccines updated to the circulating viral variants may be the optimal immunization strategy for SOT recipients in the context of a rapidly evolving pathogen.

Within the HTx recipients, multivariable analysis showed that administration of the fifth dose was associated with a higher frequency of IgG^+^ DN2 and a lower frequency of IgG^+^ DN1 BA.2^+^ RBD MBC compared to those who did not. DN2 B cells have been proposed to derive from GC-independent immune responses^30^ and DN2 to DN1 ratio has been proposed as a reflection of extrafollicular to follicular response dynamics.^35^ These findings suggest that in HTx recipients receipt of the fifth dose was associated with a stronger involvement of the extrafollicular response, possibly reflecting the activation of rapid effector responses rather than the induction of novel MBC responses.

Notably, a subset of 7 SOT recipients was categorized as low responders despite five vaccine doses, indicating that some individuals may persist as unable to effectively mount an immune response even after repeated booster doses. For these specific cases, monitoring of antibody response and alternative protective strategies should be considered.

The association between immunosuppressive drugs and vaccine immunogenicity was also explored. Consistent with previous studies,^36^^,^^37^^,^^38^ MMF treatment was associated with lower serological responses against SARS-CoV-2 in SOT recipients and with reduced frequencies of wt^+^ RBD, wt^+^BA.2^+^ RBD and BA.2^+^ RBD IgG^+^ resting MBC among HTx recipients. On the contrary, treatment with everolimus was associated with higher serological response in SOT recipients, and with higher frequencies of IgG^+^ resting RBD-specific MBC in HTx recipients. Our findings are consistent with previous studies demonstrating that in SOT recipients, everolimus-based treatment favors the development of both primary and secondary humoral immune responses, compared to the MMF-based treatment.^39^^,^^40^ Given these observations, temporarily pausing/replacing MMF treatment before vaccination might represent a potential strategy to enhance vaccine immunogenicity. However, contradictory data on the effects of MMF pausing/replacing on improving vaccine immunogenicity have been reported,^41^^,^^42^^,^^43^^,^^44^ the timing of replacement may be critical^45^ and any modification to the immunosuppressive regimen takes into account several clinical aspects which should be carefully evaluated by clinicians. Among the LuTx recipient group, no significant differences were observed in the spike-specific humoral and MBC responses between individuals undergoing different immunosuppressive regimens. However, this is likely due to the limited number of LuTx recipients included in the study, which impedes the observation of statistically significant differences. To elucidate this point, further studies conducted on larger cohorts of LuTx recipients may clarify the impact of immunosuppressive treatments.

In conclusion, the study provided an integrated characterization of the humoral and antigen-specific MBC responses elicited by SARS-CoV-2 vaccination in SOT recipients. Mathematical tools were then applied to identify clinical and demographic factors associated with differences in serological responses in SOT recipients, highlighting the value of computational analyses to profile immune responses after vaccination. These factors were also shown to be associated with differences in the MBC response. Our results indicate that the type of organ transplantation, boosting with bivalent Omicron-adapted vaccines, and immunosuppressive regimens are associated with differences in both serological and memory B cell responses induced by SARS-CoV-2 vaccination. Careful consideration of these factors is crucial to guide vaccination strategies among the HTx and LuTx recipient population, as maintaining memory B cell responses against new viral variants is a key feature of a successful vaccination strategy.

### Limitations of the study

This study has some limitations that should be considered. The number of participants differed between the HTx and LuTx recipient groups, with a limited number of LuTx recipients included in the study. Since the time of sample collection and data analysis, new Omicron-derived SARS-CoV-2 viral variants have emerged, such as JN.1 and the more recent NB.1.8.1 and XFG, which harbor more than 30 additional substitutions in the Spike protein.^46^^,^^47^^,^^48^^,^^49^ As a consequence, the immune responses here assessed may not fully represent immunity against currently circulating variants. This further underscores the importance of continuously monitoring both viral evolution and immune responses to emerging variants, particularly in populations at higher risk of developing severe forms of infection. Comparisons between samples with very high sVNT neutralizing activity are limited by signal saturation at the assay’s upper detection limit under a single fixed dilution.

The detection of RBD-specific B cells by using a single fluorophore may impact the binding specificity. To mitigate this limitation, positivity thresholds were set based on control staining performed with fluorescent streptavidin alone. Also, the relatively small sample size and number of antigen-specific cells detected could have affected the phenotypic analyses, limited the statistical robustness and increasing the noise of MBC subset comparisons. Therefore, the evaluation of MBC subsets should be considered exploratory and hypothesis-generating. Finally, the study includes only participants who received mRNA vaccines and not other vaccine formulations and, due to limited blood sample availability, the T cell response has not been investigated. Previous studies in SOT recipients have shown that mRNA vaccines can induce CD4^+^ and CD8^+^ T cell responses, although weaker than in immunocompetent individuals, which may increase after boosters.^50^^,^^51^ However, T cell reactivity against Omicron variants, including BA.4/5, was found to be preserved even in SOT recipients with limited neutralizing antibody response, suggesting a potential role of cellular immunity in protection in this population.^52^ Future studies involving larger cohorts and integrating B and T cell immune profiling will be important to better define vaccine responses in SOT recipients.

## Resource availability

### Lead contact

Requests for further information and resources should be directed to and will be fulfilled by the lead contact Annalisa Ciabattini (annalisa.ciabattini@unisi.it).

### Materials availability

No unique reagents were generated in this study.

### Data and code availability

• All the data supporting the findings of this study are presented within the manuscript. The dataset generated during the current study has been deposited in the Zenodo data repository and is available upon reasonable request of access (Zenodo Data: https://doi.org/10.5281/zenodo.21699915). The repository includes anonymized immunological and clinical data used for the analyses described in the manuscript. The original code underlying the dimensionality reduction and unsupervised clustering analyses of the serological data reported in this study is publicly available in the Zenodo data repository (Zenodo data: https://zenodo.org/records/17466772).
• Any additional information required to reanalyse the data reported in this paper is available from the lead contact upon request.

## Acknowledgments

We would like to thank the Nursing staff of the clinics for collecting blood. We thank all the volunteers and the individuals who participated to the study. The graphical abstract was created in BioRender. Coppola, C. (2026) https://BioRender.com/oww2kr1. Funding: This work was supported by Piano Nazionale di Ripresa e Resilienza (PNRR) PE13 INF_ACT funded by Unione Europea – Next Generation EU (Project Number: PE00000007) and by the Department of Medical Biotechnologies of the 10.13039/100012990University of Siena (D.M.).

## Author contributions

Conceptualization: A.C., D.M., F.M., M.F., S.B., and D.B.; patients and volunteers enrollment: F.M., M.F., S.B., and D.B.; data curation: C.C., A.L., F.P., S.B., and D.B.; formal analysis: C.C., S.C., G.M., and S.L.; funding acquisition: D.M.; investigation: C.C., S.C., J.P., G.M., and S.L.; resources: D.M.; software: G.M. and S.L.; supervision: A.C. and D.M.; visualization: A.C., C.C., S.C., G.M., and S.L.; writing – original draft: C.C., S.C., and A.C.; writing – review and editing: all the authors reviewed and edited the final version of the manuscript.

## Declaration of interests

The authors declare no competing interests.

## STAR★Methods

### Key resources table

**Table:** 

REAGENT or RESOURCE	SOURCE	IDENTIFIER
**Antibodies**		

Goat Anti-Human IgG-HRP	SouthernBiotech	Cat# 2040-05: RRID: AB_2795644
SARS-CoV-2 (2019-nCoV) Spike Neutralizing Antibody, Rabbit Mab	Sino Biological	Cat# 40592-R118; RRID: AB_3677186
BD Pharmingen™ Human BD Fc Block™	BD Biosciences	Cat# 564220; RRID:AB_2869554
BD Horizon™ Fixable Viability Stain 700	BD Biosciences	Cat# 564997; RRID:AB_2869637
BD OptiBuild™ BV650 Mouse Anti-Human CD3	BD Biosciences	Cat# 750984; RRID:AB_2875053
BD Horizon™ BV605 Mouse Anti-Human CD19	BD Biosciences	Cat# 562653; RRID:AB_2722592
BD Horizon™ PE-CF594 Mouse Anti-Human IgD	BD Biosciences	Cat# 562540; RRID:AB_11153129
BD OptiBuild™ BV786 Mouse Anti-Human CD27	BD Biosciences	Cat# 751676; RRID:AB_2875662
BD OptiBuild™ BV711 Mouse Anti-Human IgM	BD Biosciences	Cat# 740795; RRID:AB_2740458
BD Pharmingen™ APC-H7 Mouse Anti-Human CD20	BD Biosciences	Cat# 560734; RRID:AB_1727449
BD Pharmingen™ FITC Mouse Anti-Human CD21	BD Biosciences	Cat# 561372; RRID:AB_10895576
BD OptiBuild™ BB700 Mouse Anti-Human CD11c	BD Biosciences	Cat# 748270; RRID:AB_2872698
BD Horizon™ BUV395 Mouse Anti-Human CD38	BD Biosciences	Cat# 563811; RRID:AB_2744372
BD Pharmingen™ PE-Cy™7 Mouse Anti-Human IgG	BD Biosciences	Cat# 561298; RRID:AB_10611712
IgA Antibody, anti-human, VioGreen™	Miltenyi Biotech	Cat# 130–113–481; RRID:AB_2734099

**Biological samples**		

Human blood samples	Samples were collected after informed consent from participants enrolled in the observational and longitudinal PatoVac_COV and IMMUNO_COV studies, conducted at the Siena University Hospital, Siena, Italy. The studies were performed in compliance with all relevant ethical regulations and the protocols were approved by local Ethical Committee for Clinical experimentation of Regione Toscana Area Vasta Sud Est (CEAVSE; protocol code n.19479, approved on 15^th^ March 2021 for the PatoVac study and protocol code n.18869, approved on 21^st^ December 2020 for the IMMUNO_COV study).	–

**Chemicals, peptides, and recombinant proteins**		

Lymphoprep™	STEMCELL Technologies	Cat# 07861
SARS-CoV-2 (2019-nCoV) Spike S1+S2 ECD-His Recombinant Protein	Sino Biological	Cat# 40589-V08B1
SARS-CoV-2 (BA.2) Spike S1+S2 trimer Protein (ECD, His Tag) (HPLC-verified)	Sino Biological	Cat# 40589-V08H28
SARS-CoV-2 Spike protein RBD-HRP, BA.2 variant, His Tag	GenScript	Cat# Z03741
Biotinylated Recombinant SARS-CoV-2 S Protein RBD (carrier-free)	Biolegend	Cat# 793906
Biotinylated SARS-CoV-2 Spike RBD Protein, His,Avitag™ (BA.2/Omicron) (MALS verified)	Acrobiosystems	Cat# SPD-C82Eq-25 μg
BD Horizon™ BV421 Streptavidin	BD Biosciences	Cat# 563259
BD Horizon™ BUV737 Streptavidin	BD Biosciences	Ca# 612775
BD Horizon™ Brilliant Stain Buffer	BD Biosciences	Cat# 563794
BD Cytofix™ Fixation Buffer	BD Biosciences	Cat# 554655

**Critical commercial assays**		

SARS-CoV-2 Surrogate Virus Neutralization Test Kit	GenScript	Cat# L00847-A

**Software and algorithms**		

REDCap	Research Electronic Data Capture, Vanderbilt University	https://project-redcap.org/
GraphPad Prism v10	GraphPad Software	https://www.graphpad.com/scientific-software/prism/
FlowJo v10	TreeStar, Inc	https://www.flowjo.com/
R v.4.3.3	The R Project	https://www.r-project.org/
Original dataset generated in this study	Coppola et al.^53^	Zenodo Data: https://doi.org/10.5281/zenodo.21699915
Rtsne v0.17	Krijthe^54^	N/A
Uwot v0.2.3	Melville et al.^55^	N/A
mclust library v6.1.1	Scrucca et al.^56^	N/A
Original code generated in this study	Coppola et al.^57^	Zenodo Data: https://doi.org/10.5281/zenodo.17466772
FlowSOM package v2.4.0	Van Gassen et al.^58^	N/A
flowDensity package v1.34.0	Malek et al.^59^	N/A
MatchIt v4.7.2	Ho et al.^60^	N/A
Glmnet package v4.1-8	Friedman et al.^61^	N/A

### Experimental model and study participant details

#### Study population

A total of 117 SOT recipients and 38 HC as controls were enrolled in the observational longitudinal PatoVac_COV and IMMUNO_COV studies, respectively, conducted at the Siena University Hospital, Siena, Italy. The studies were performed in compliance with all relevant ethical regulations and the protocols were approved by local Ethical Committee for Clinical experimentation of Regione Toscana Area Vasta Sud Est (CEAVSE; protocol code n.19479, approved on 15^th^ March 2021 for the PatoVac_COV study and protocol code n.18869, approved on 21^st^ December 2020 for the IMMUNO_COV study).

SOT recipients were recruited at the Respiratory Disease and Lung Transplant Unit, and at Cardiac Surgery Unit, Azienda Ospedaliera Universitaria Senese. HC were recruited at the Infectious and Tropical Diseases Unit, Azienda Ospedaliera Universitaria Senese through an information campaign targeting citizens, health care workers and academic staff. All participants provided a written informed consent before participation in the study. Inclusion criteria were age ≥18 years and adherence to the COVID-19 vaccination campaign (for both studies), previous heart or lung transplantation and at least one SARS-CoV-2 vaccination after organ transplant (for the PatoVac_COV study). Exclusion criteria were pregnancy, withdrawal of consent or refusal to participate (for both studies) and immunocompromising comorbidities (congenital, acquired, or drug-related for the IMMUNO_COV study).

Vaccine administration was performed according to the Italian vaccination program. All participants received the first two doses of BNT162b2 (Comirnaty; Pfizer-BioNTech), or mRNA-1273 (Spikevax, Moderna) vaccines, administered 21 and 28 days apart, respectively, according to the national guidelines. Third, fourth and fifth vaccine doses were administered at 5–7 months, 12–18 months and 18–21 months from the first vaccine dose, respectively. All doses administered until October 2022 were performed with the wt monovalent vaccines, vaccine doses administered afterward may include either the wt monovalent or bivalent Omicron-adapted vaccine formulations (bivalent wt/Omicron BA.1, bivalent wt/Omicron BA.4-5). SOT recipients and HC provided infection-related information when they were called for blood collection. SARS-CoV-2 infection among HC was assessed through passive surveillance. Participants completed two surveys (February 2022 and May 2023) and updated their infection status at each blood collection visit. Infection was defined based on symptoms or confirmed exposure and verified by antigen or molecular testing on nasopharyngeal swabs, either self-administered or collected by healthcare professionals. Participants reported the date of their positive test. For SOT participants, infection-related information, including the date of a positive antigen or molecular test, was collected directly by clinicians at each blood draw. Tests were performed on nasopharyngeal swabs, either self-administered or collected by healthcare professionals. Infections reported from December 2021 onward were assumed to be due to the Omicron variant, which was predominant in Italy at that time. REDCap software software (Vanderbilt University, Nashville, TN, USA) was employed for the collection and management of clinical data. Details on demographic, clinical, SARS-CoV-2 vaccination and infection data are reported in the results section. The dataset generated during the current study is available in the Zenodo data repository (Zenodo Data: https://doi.org/10.5281/zenodo.21699915).^53^

### Method details

#### Blood sample collection

Blood samples were collected in heparin-coated blood tubes (BD Vacutainer, USA) between May and September 2023, two years after the first vaccine dose. PBMCs were isolated by density-gradient sedimentation, using Ficoll-Paque (Lymphoprep, STEMCELL Technologies, Canada). PBMCs were cryopreserved in liquid nitrogen. Plasma samples were stored at −80°C. Plasma samples were heat-treated for 1h at 56°C to inactivate potential pathogens and complement proteins that could interfere with antibody detection.

#### Enzyme-linked immunosorbent assay

IgG antibodies against full spike protein (S1+S2 ECD) of wt and Omicron BA.2 variant were assessed as previously described.^62^ Briefly, microtitre plates were coated with 1 μg/mL of wt or BA.2 full spike protein (S1+S2 ECD, both from Sino Biological, China), blocked and incubated with serial dilution of heat-inactivated plasma samples. Anti-human horseradish peroxidase (HRP)-conjugated IgG was added, and plates were developed with 3,3′,5,5′-Tetramethylbenzidine (TMB; Thermo Fisher Scientific). A SARS-CoV-2 Spike neutralizing antibody (SARS-CoV-2 [2019-nCoV] Spike Neutralizing Antibody, Rabbit Mab, Sino Biological) was added and titrated in each plate, to obtain a standard calibration curve. The absorbance was measured at 450 nm using a Multiskan FC Microplate Photometer (Thermo Fisher Scientific). Data are expressed as ng/ml.

#### ACE2/RBD inhibition assay

ACE2/RBD binding inhibition against the wt strain and the Omicron BA.2 RBD variant were tested with a SARS-CoV-2 sVNT kit (cPass, Genscript), according to the manufacturer protocol. Briefly, plasma samples were diluted 1:20 and incubated with HRP-wt RBD or HRP-BA. 2 RBD for 30 min, 37°C. Mixtures were added to human ACE2 pre-coated wells and incubated for 15 min, RT. After substrate addition and development for 15 min RT, the absorbance was measured at 450 nm on a Multiskan FC Microplate Photometer (Thermo Fisher Scientific). Inhibition values ≥30% were considered as positive results, and values <30% as negative results, as previously established^63^ and indicated by the manufacturer.

#### Clustering analysis of serological data

Log_2_-transformed IgG concentrations underwent *Z* score scaling alongside ACE2/RBD binding inhibition capacity against wild-type and Omicron BA.2, yielding a standardized four-dimensional feature space. Eight clustering workflows were evaluated by combining two algorithms—k-means and Gaussian Mixture Modeling (GMM)—across four data representations: original 4D standardized space, PCA-reduced space, t-SNE embedding (perplexity = 38) and UMAP embedding (n_neighbors = 15, min_dist = 0.1). Dimensionality reduction was carried out using Rtsne v0.17 (t-SNE)^54^ and uwot v0.2.3 (UMAP).^55^ Clustering was performed using statskmeans (nstart = 20) and mclust v6.1.1.^56^ Cluster compactness and separation were evaluated via within-cluster sum of squares (WSS) and average mean silhouette width (AWS), computed using the cluster package. The optimal number of clusters for k-means was chosen by maximization of AWS across k = 2–10. GMM components were selected to minimize Bayesian Information Criterion (BIC). WSS and AWS were used to select the best performing clustering algorithm. For the Original 4D and PCA-reduced spaces, cluster boundaries were projected onto the first two principal components (PC1–PC2) of the standardized data; t-SNE and UMAP solutions were displayed in their respective two-dimensional embedding spaces. The complete description of the clustering analysis is reported in the supplemental material (Methods S1). The complete analysis code is publicly available in the Zenodo data repository (Zenodo Data: https://zenodo.org/records/17466772.^57^) All analyses were performed in R v4.5.0.

#### Multiparametric flow cytometry

RBD-specific B cells were detected using biotinylated antigens in combination with different streptavidin (SA)–fluorophore conjugates. Biotinylated RBD antigens were tetramerized with SA-fluorophore conjugates, added in 5 steps, 20′ apart, at 4°C under shaking. Biotinylated wt RBD (BioLegend) and biotinylated Omicron BA.2 RBD (ACROBiosystems) were tetramerized with SA-BV421 and SA-BUV737 (both from BD Biosciences) respectively, at a 4:1 M ratio. For staining, approximately 2 × 10^6^ PBMCs were incubated with the BD human Fc block (BD Bioscience) for 10 min at RT, labeled with Live/Dead FVS700 (BD Bioscience) for 10 min at RT and stained with a mix containing 100 ng of each wt RBD-BV421 and BA.2 RBD-BUV737 tetramers, for 1 hour at 4°C. Thresholds for identifying RBD-specific MBC were defined using streptavidin-only controls to exclude nonspecific binding (Figure S4). Nonspecific binding of the BV421- and BUV737 antigen baits was assessed using an unrelated antigen (Figure S4). Subsequently, cells were stained for 30 min at 4°C with the following antibody cocktail in Brilliant buffer (BD biosciences): CD3-BV650 (clone OKT3, dilution 1:200), CD19-BV605 (clone SJ25C1, dilution 1:50), IgD-PE-CF594 (clone IA6-2, dilution 1:100), CD27-BV786 (clone O323, dilution 1:50), IgM-BV711 (clone G20-127, dilution 1:100), CD20-APC-H7 (clone 2H7, dilution 1:100), CD21-FITC (clone B-ly4, dilution 1:50), CD11c-BB700 (clone 3.9, dilution 1:50), CD38-BUV395 (clone HB7, dilution 1:200), IgG-PE-Cy7 (clone G18-145, dilution 1:100) (all from BD Bioscience) and IgA-VioGreen (clone IS11-8E10, dilution 1:100, Miltenyi Biotec). Following surface staining, cells were washed once with PBS, fixed in BD fixation solution (BD Biosciences) and acquired with the LSRFortessa X20 flow cytometer (BD Biosciences). Data analysis was performed on sample containing at least 1000 total B cells using FlowJo v10 (TreeStar, USA) and with computational FlowSOM algorithm, as described below.

#### Unsupervised flow cytometry analysis

The MBC population was identified as live, singlet, CD3^-^/CD19^+^ cells, excluding from the analysis the naive B cells (CD27^-^/IgD^+^), using FlowJo v10 (TreeStar, USA). FCS files were imported into R environment, compensated and transformed as previously described.^64^ All fluorescence were visualized across all samples to assess potential batch effects and plotted against the time parameter to exclude potential anomalies or drifts during data acquisition. Clustering analysis was performed following the FlowSOM function pipeline (FlowSOM package v2.4.0).^58^ SOM grid size was set to 10 × 10. Similar nodes were merged into 20 metaclusters (metaclustering step) and similar metaclusters were manually merged. The Euclidean distance was used in both clustering and metaclustering steps. Thresholds to bisect positive and negative cells for each marker expression were automatically set with the flowDensity package v1.34.0.^59^ FlowSOM results were displayed as a heatmap reporting the percentage of positive cells for each marker within the metacluster. FlowJo gates were imported in R environment using GetFlowJoLabels function to calculate the frequencies of antigen-specific cells within each cluster. Cluster frequency analyses were performed separately for wt^+^, wt^+^BA.2^+^, and BA.2^+^ RBD-specific MBC, only in samples with at least five cells in the RBD-specific MBC population. Frequencies of unsupervised clusters significantly differing between groups were validated against manual gating by Spearman correlation analysis (Figures S5–S7).

### Quantification and statistical analysis

Variables were reported as medians with interquartile ranges (IQR) as measure of dispersion, and binary variables were reported as counts and percentages. Non-parametric statistical tests were employed in this study, as not all features (i.e., antibody concentrations and antigen specific B cell frequencies) followed a Gaussian distribution, as reported by visual inspection (Q-Q plots and histograms). Tests were also two-tailed, as no prior assumptions were made before the analyses.

Age and sex were matched for comparing humoral response between SOT recipients and HC. Propensity score was performed using the MatchIt package (v4.7.2).^60^ Matching was conducted separately by sex using a 1:1 nearest neighbor algorithm with a caliper of 0.2, considering age as the matching covariate to balance the cohorts.

Statistical significance between groups were assessed using Mann-Whitney and Kruskal–Wallis tests. Dunn’s post-test was applied following Kruskal–Wallis test for multiple comparisons. Differences in the number of participants positive for the ACE2/RBD binding inhibition between SOT recipient group and HC were assessed using Fisher’s exact test.

Mann-Whitney and Fisher’s exact tests were used for comparing continuous and categorical variables respectively, between the clusters defined by the k-means clustering algorithm.

To complement the univariable comparisons of clinical and demographic characteristics across responder groups, a penalized multinomial logistic regression model was fitted using the Least Absolute Shrinkage and Selection Operator (LASSO). Variables with near-zero variance, defined as those with fewer than 5% of samples in either category, were excluded prior to model fitting. The analysis was performed in R using the glmnet package (v4.1-8).^61^ LASSO regularization (alpha = 1) was applied to perform feature selection by shrinking the coefficients of less informative variables to zero. The optimal value of the regularization parameter (lambda) was determined using the c v.glmnet function with k-fold cross-validation (k = 10), based on multinomial deviance. The lambda value corresponding to lambda min was selected for the final model. The statistical significance of the model was assessed using a permutation-based approach (*n* = 10000 permutations), in which cluster labels were randomly shuffled to generate a null distribution of model performance. The observed performance (measured as accuracy) was compared against this null distribution to derive a *p*-value.

To complement the univariable comparisons of RBD-specific MBC subset frequencies, GLM with binomial distribution were fitted using the glm function in R. The following variables were included as predictors: age, sex, immunosuppressive treatment, and fifth dose status. Analyses were performed separately for each cohort, as well as on the combined dataset including cohort as an additional variable. Only clusters with a mean frequency of antigen-specific cells ≥10% across samples were included in the analysis. For each selected cluster, the outcome variable was defined by dichotomizing the frequency of antigen-specific cells at the median value across samples. This dichotomization was applied to reduce the impact of skewed distributions and improve model interpretability. Statistically significant *p* values are reported as numeric values in the results section and as asterisks on the graphs (∗*p* < 0.05, ∗∗*p* < 0.01, ∗∗∗*p* < 0.001). Statistical details of experiments can be found in the figure legends. All analyses were carried out using R v4.5.0 environment for statistical computing (R Foundation for Statistical Computing, Vienna, Austria) and GraphPad Prism v9 (GraphPad Software, San Diego, CA, USA).
