# Supplementary material for: Memory B cell response to SARS-CoV-2 vaccination in people with heart and lung transplantation: Identification of associated clinical factors
Source: iScience. 2026 Sep 7;29(9):117428. doi: 10.1016/j.isci.2026.117428 (PMC13577873; doi:10.1016/j.isci.2026.117428)
Supplement: Supplementary file 1 [file mmc1.pdf]

iScience, Volume *29*

Supplemental information

Memory B cell response to SARS-CoV-2 vaccination in people with heart and lung transplantation: Identification of associated clinical factors

Chiara Coppola, Simone Costagli, Giorgio Montesi, Simone Lucchesi, Jacopo Polvere, Arianna Lippi, Fabio Pierguidi, Francesca Montagnani, Massimiliano Fabbiani, Sonia Bernazzali, David Bennett, Donata Medaglini, and Annalisa Ciabattini

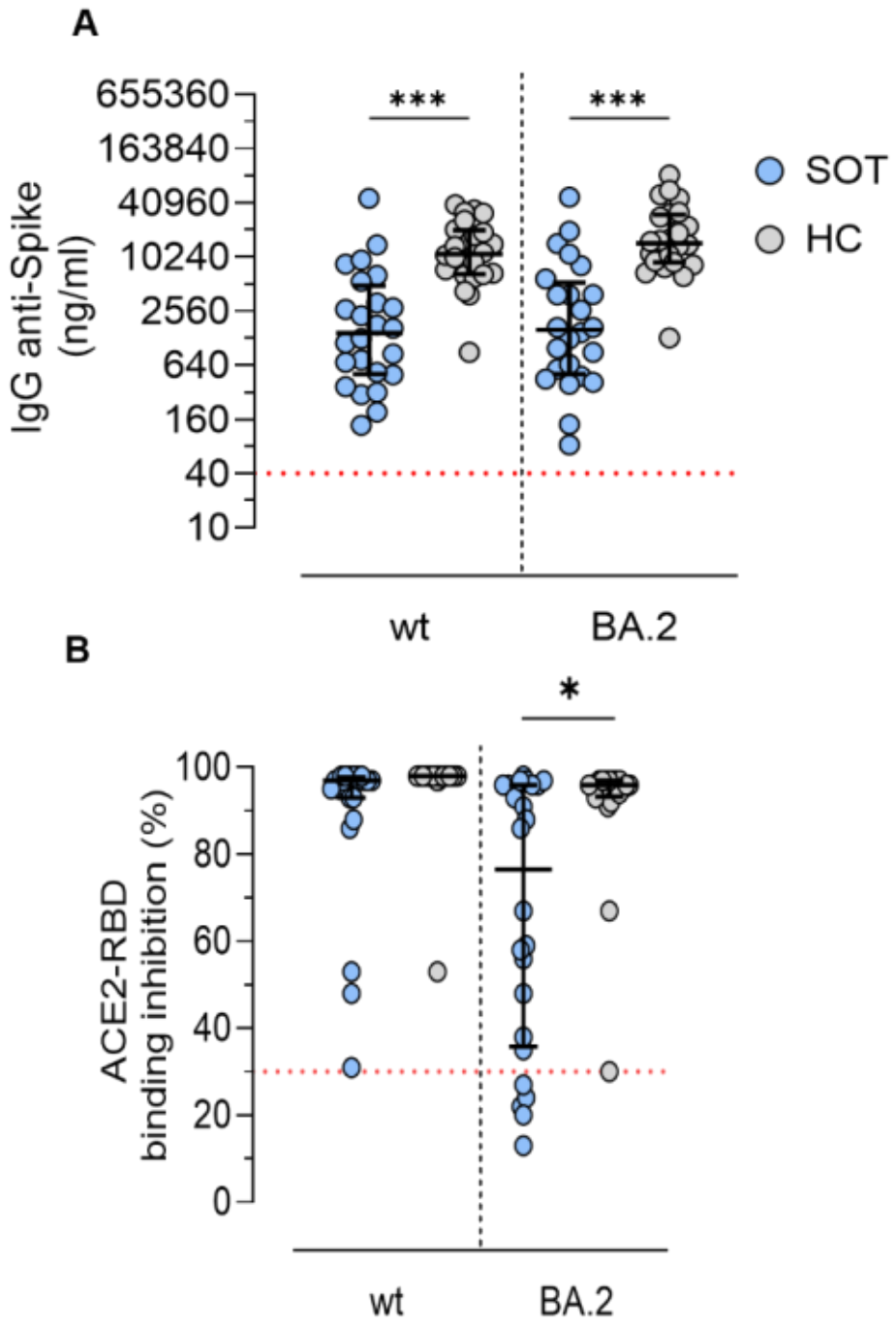

Figure S1. Characterization of the humoral response in age- and sex-matched SOT recipients and HC subgroup, related to Figure 2. A) Spike-specific IgG targeting the wt strain and the Omicron BA.2 variant (BA.2) were assessed in the solid organ transplant recipients (SOT) and healthy controls (HC), adjusted for age and sex. Values are reported as antibody concentration (ng/ml). A threshold (dotted red line) was placed at the pre-pandemic cut-off for wt spike-specific IgG to discriminate between seropositive and seronegative samples. B) Antibody capacity to inhibit the ACE2/RBD binding for the wt strain and the Omicron BA.2 variant, assessed by sVNT. A threshold (dotted red line) was placed at 30% inhibition percentage to discriminate between positive and negative samples. *$p < 0.05$, ***$p < 0.001$. Sample size: SOT (A-B: n=24); HC (A-B: n=24).

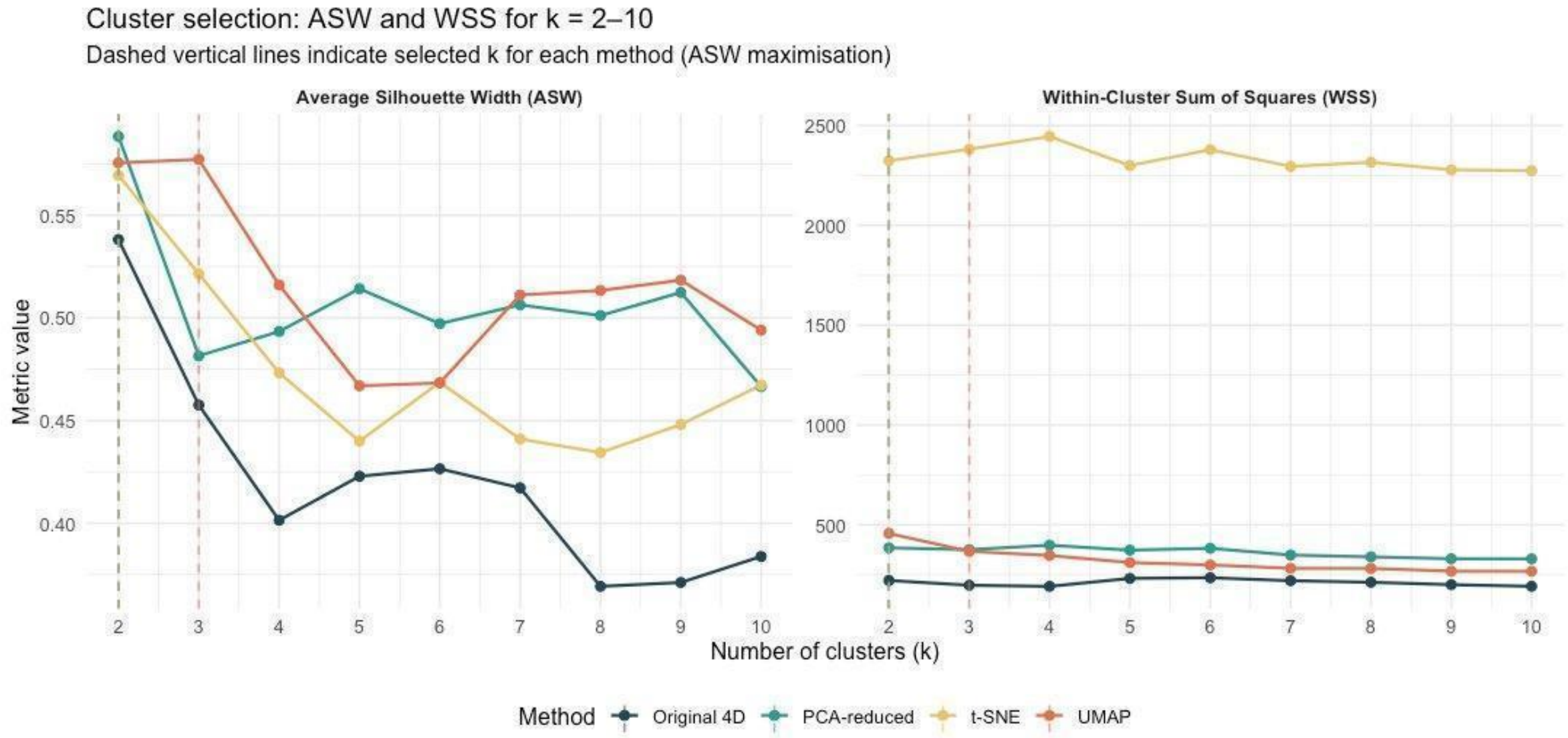

Figure S2. Metrics of k-means clustering as a function of the number of clusters k (2–10), related to STAR Methods. Average Silhouette Width (ASW; left) and Within-Cluster Sum of Squares (WSS; right) as a function of the number of clusters k (2–10) for k-means clustering applied to four data representations: Original 4D standardized space (dark blue), PCA-reduced space (teal), t-SNE embedding (yellow), and UMAP embedding (orange/red). Dashed vertical lines indicate the k selected by ASW maximization for each method. Note that t-SNE WSS values operate on a different numerical scale and are not directly comparable with those of the remaining methods.

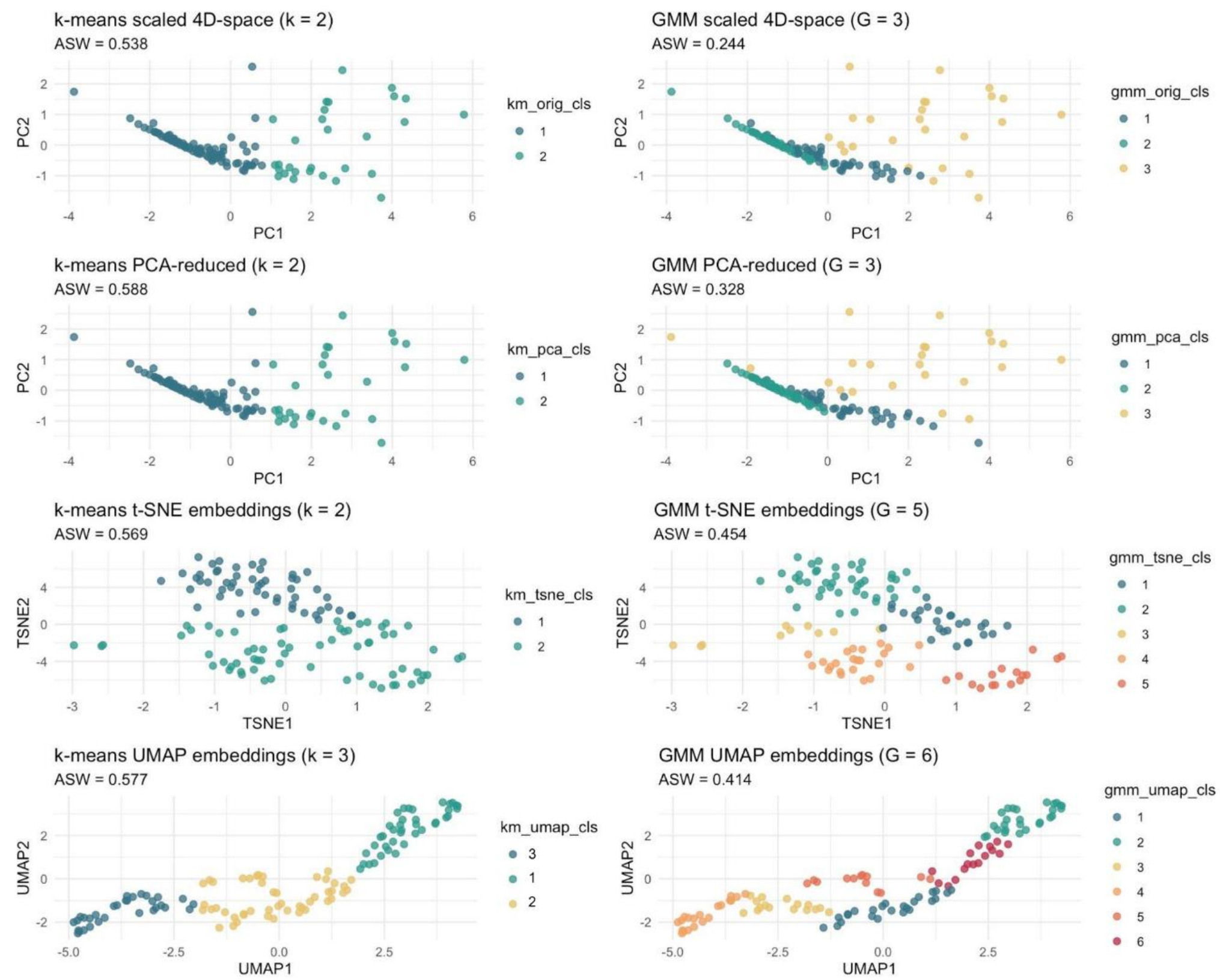

Figure S3: Unsupervised Clustering of serological data, related to Figure 3. Clustering of four z-scored immunological features, antigen-specific IgG concentrations and neutralization against SARS-CoV-2 wild-type and Omicron BA.2, using k-means and Gaussian mixture model (GMM) across four embedding spaces. For the Original 4D and PCA-reduced spaces, cluster boundaries are projected onto the first two principal components (PC1–PC2) of the standardized data; t-SNE and UMAP solutions are displayed in their respective two-dimensional embedding spaces. Top row: k-means (left) and GMM (right) applied to the Original 4D standardised space, projected onto PC1 and PC2. Second row: k-means (left) and GMM (right) applied to the PCA-reduced space. Third row: k-means (left) and GMM (right) applied to the t-SNE embedding. Bottom row: k-means (left) and GMM (right) applied to the UMAP embedding. Each point represents an individual sample, coloured by its assigned cluster. ASW for each solution is reported in the panel title.

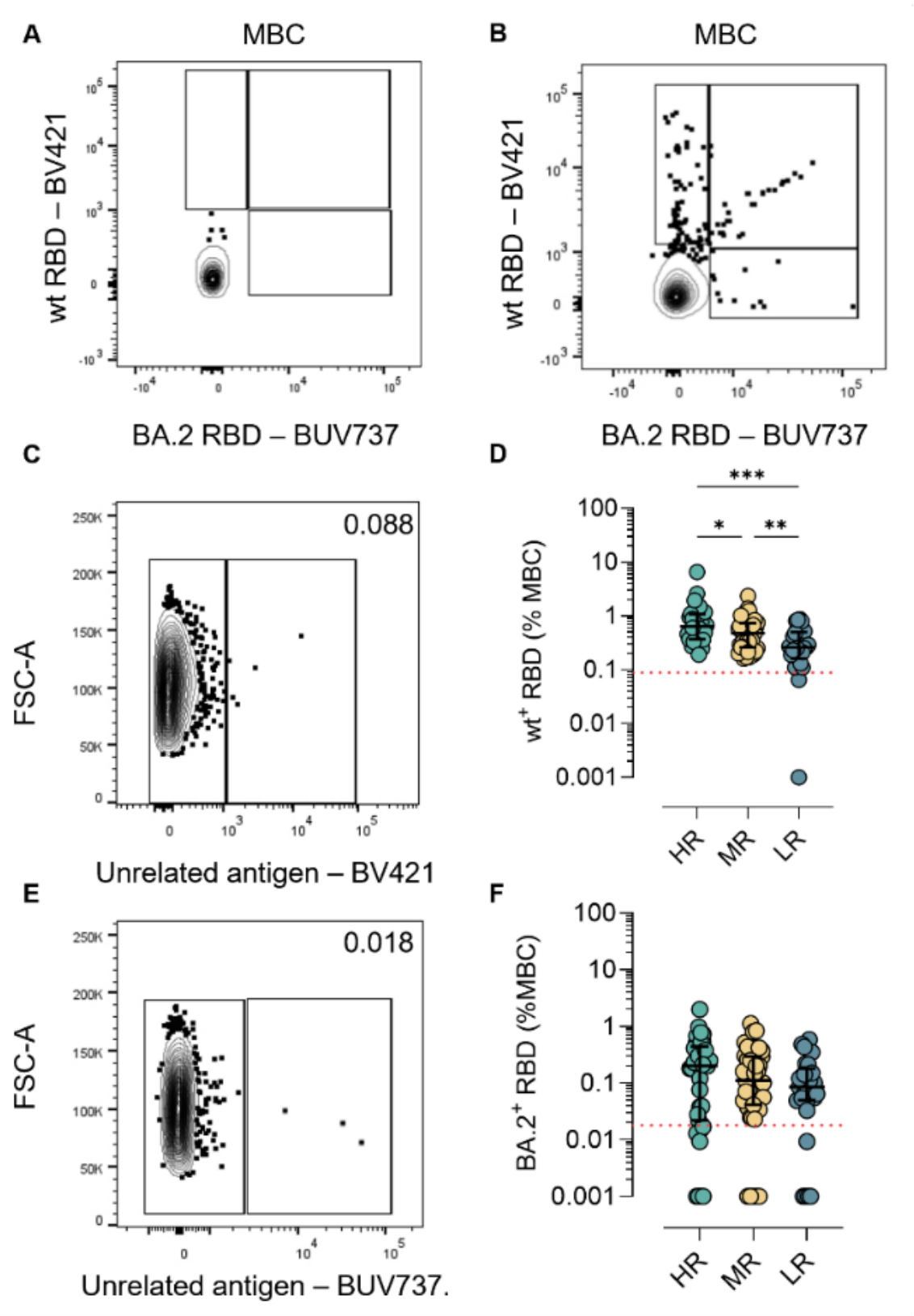

Figure S4: Streptavidin alone and mock control staining, related to STAR Methods. A) Representative dot plots showing B cells stained with BV421 and BUV737-conjugated streptavidins without (A) or with (B) preincubation with biotinylated RBD antigen. C-F) Dot plots from two concatenated PBMCs samples stained with streptavidin-BV421 (C) or streptavidin-BUV737 (E) preincubated with an unrelated biotinylated antigen. The non-specific binding values (top right) are reported as thresholds (dotted line) in panel D and F showing the frequency of wt^+ and $BA.2^+$ RBD MBC, respectively. *$p < 0.05$, **$p < 0.01$, ***$p < 0.001$.

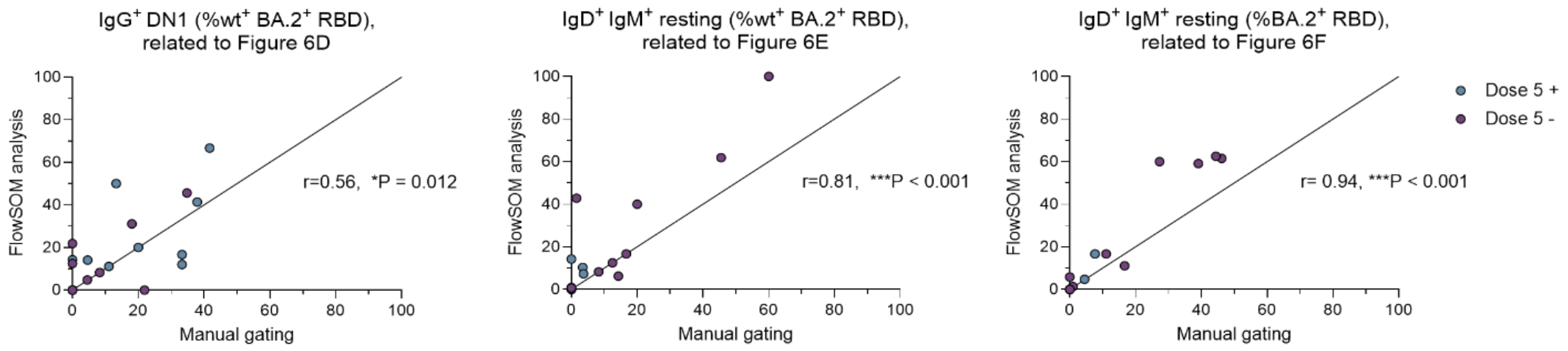

Figure S5. Validation of FlowSOM-identified MBC subsets by correlation with manual gating frequencies in LuTx recipients stratified by the administration of the fifth, bivalent vaccine dose, related to STAR Methods and Figure 6. Spearman correlation between frequencies obtained by FlowSOM analysis and manual gating was performed to validate FlowSOM results for subset significantly different between groups. A diagonal line represents the x = y equation. *$p < 0.05$, ***$p < 0.001$.

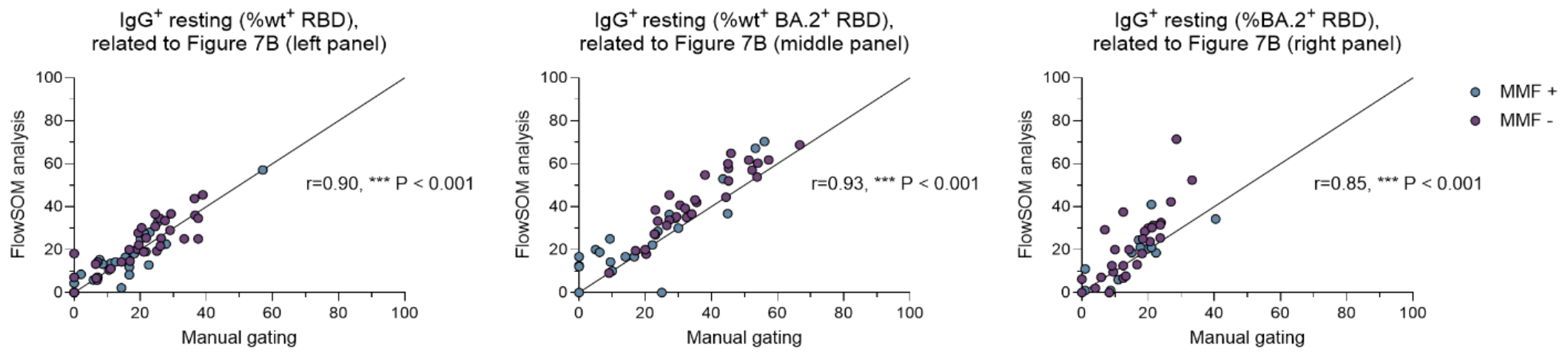

Figure S6. Validation of FlowSOM-identified MBC subsets by correlation with manual gating frequencies in HTx recipients stratified by MMF treatment, related to STAR Methods and Figure 7. Spearman correlation between frequencies obtained by FlowSOM analysis and manual gating was performed to validate FlowSOM results for subset significantly different between groups. A diagonal line represents the x = y equation. ***$p < 0.001$.

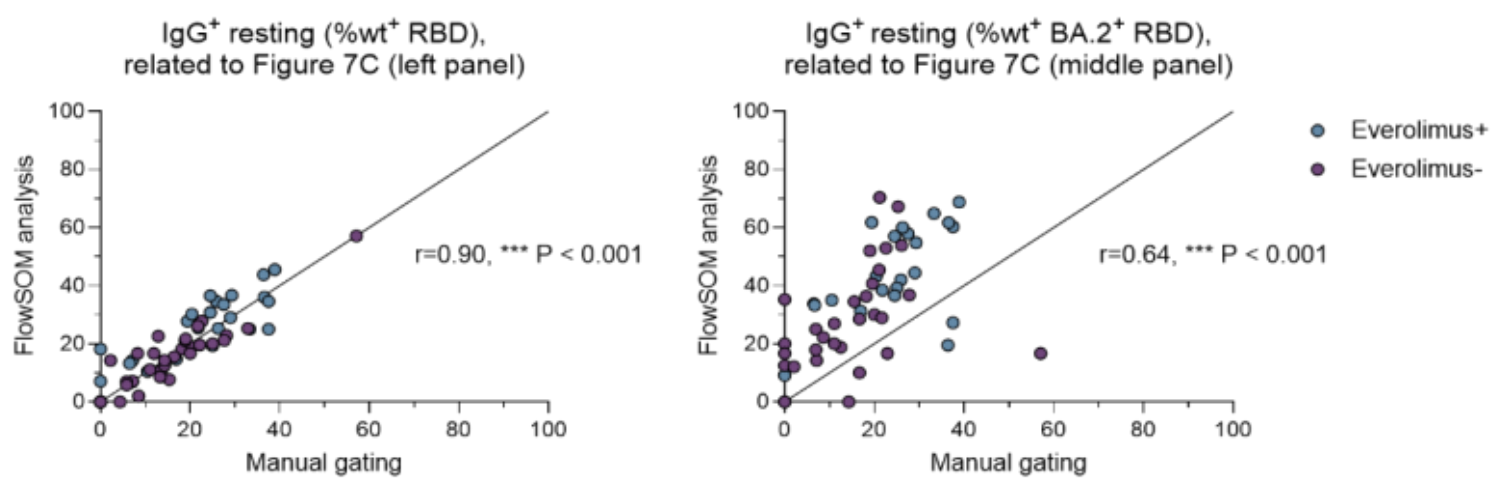

Figure S7. Validation of FlowSOM-identified MBC subsets by correlation with manual gating frequencies in HTx recipients stratified by everolimus treatment, related to STAR Methods and Figure 7. Spearman correlation between frequencies obtained by FlowSOM analysis and manual

gating was performed to validate FlowSOM results for subset significantly different between groups. A diagonal line represents the x = y equation. ****p* < 0.001.

Table S1. Metrics of clustering methods, related to Figure 2. Summary of clustering performance metrics for all eight workflows. The selected method (k-means on UMAP embedding, k = 3) is shown in bold.

Clustering method	k / G	WSS	ASW
k-means, Original 4D space (k = 2)	2	221	0.538
GMM, Original 4D space (G = 3)	3	249	0.244
k-means, PCA-reduced space (k = 2)	2	385	0.588
GMM, PCA-reduced space (G = 3)	3	356	0.328
k-means, t-SNE embedding (k = 2)	2	2,324	0.569
GMM, t-SNE embedding (G = 5)	5	2,428	0.454
**k-means, UMAP embedding (k = 3)**	**3**	**367**	**0.577**
GMM, UMAP embedding (G = 6)	6	357	0.414

WSS= Within-Cluster Sum of Squares; ASW= Average Silhouette Width; k / G= number of clusters or Gaussian components identified.

Table S2. Contingency table of subject-level cluster assignments between k-means on the Original 4D standardised space (rows, k = 2) and k-means on the UMAP embedding (columns, k = 3), related to STAR Methods.

	UMAP cluster 1 (HR)	UMAP cluster 2 (MR)	UMAP cluster 3 (LR)
**Original 4D cluster 1 (HR + MR)**	39	46	3
**Original 4D cluster 2 (LR)**	0	1	28

HR= high responders; MR= medium responders; LR= low responders.

Table S3. LASSO regression coefficients for clinical and demographic variables associated with humoral vaccine responsiveness, related to Table 3 and Figure 4. A Lasso model was fitted to identify variables independently associated with low humoral vaccine responsiveness in the multivariable setting. Non-relevant variables in the LASSO model are reported with a coefficient of zero. Variables with near-zero variance (< 5% of samples in either category) were excluded prior to model fitting.

Clinical variables	LASSO coefficients		
	LR categorisation	MR categorisation	HR categorisation
Treatment with MMF [a]	0.07	0	0
Treatment with tacrolimus [a]	0	0	0
Treatment with prednisone [a]	0	0	0
Treatment with cyclosporine [a]	0	0	0
Treatment with everolimus [a]	-0.79	0	0
Time since transplant	0	0	0
Infection post 4th dose [a]	0	0	0
Infection post 3rd dose [a]	0	0.50	0
Last antigen exposure	0	0	-0.01
5th dose [a]	-0.27	0	0
4th dose [a]	0	0	0
Type of organ transplant [b]	0.08	0	-0.14
Sex [c]	-0.06	0	0
Age	0	-0.01	0

HR= high responders; MR= medium responders; LR= low responders. [a] For variables with a yes/no condition, the value 0 and 1 were assigned to individuals that have the condition "no" and "yes", respectively. [b] For the "type of organ transplant" variable the value 0 and 1 were assigned to the condition "heart transplant" and "lung transplant", respectively. [c] For the "sex" variable the value 0 and 1 were assigned to the condition "female" and "male", respectively.

Table S4. Median of each MBC subset among the wt$^+$ RBD, wt$^+$BA.2$^+$ RBD and BA.2$^+$ RBD specific MBC, related to Figure 6. The median frequencies reported refer to each respective parental population (wt$^+$ RBD, wt$^+$BA.2$^+$ RBD and BA.2$^+$ RBD specific MBC)

	Cluster 1	Cluster 2	Cluster 3	Cluster 4	Cluster 5	Cluster 6	Cluster 7	Cluster 8	Cluster 9	Cluster 10	Cluster 11	Cluster 12	Cluster 13	Cluster 14	Cluster 15	Cluster 16	Cluster 17
**In wt$^+$ RBD**																	
HTx recipients (dose 5+)	17.39	2.06	4.35	0	3.23	0	0	0	0	19.44	2.25	0	0	14.67	4.55	0	0
HTx recipients (dose 5-)	13.18	3.79	2.64	0	1.96	1.76	0	0	0	17.87	1.43	0	0	18.55	4.88	0	0
LuTx recipients (dose 5+)	7.07	0	0.93	0.93	6.45	0	2.93	0	0	28.17	0.79	0	0	21.86	3.31	0	0
LuTx recipients (dose 5-)	5.71	0	2.33	0	0	0.3	0	0	0	22.22	2.33	0	0	33.33	4.35	0	0
**In wt$^+$ BA.2$^+$ RBD**																	
HTx recipients (dose 5+)	2.08	0	0.76	0	1.82	0	0.72	0	0	27.27	0.72	0	0	33.77	4.76	0	0
HTx recipients (dose 5-)	2.87	0	2.17	0	2.75	0	0	0	0	25	0.39	0	0	35.15	0.87	0	0
LuTx recipients (dose 5+)	0	0	0	0	3.45	0	3.45	0	0	16.67	0	0	0	34.48	0	0	0
LuTx recipients (dose 5-)	14.58	0	0	0	0.36	0	0.78	0	0	6.55	0	0	0	35.22	0	0	0
**In BA.2$^+$ RBD**																	
HTx recipients (dose 5+)	0	0	0	0	6.45	0	0	0	0	20	25	0	0	17.5	4.35	0	0
HTx recipients (dose 5-)	1.59	0	0	0	2.63	2.38	0.93	0	0	28.57	9.8	0	0	20	0	0	0
LuTx recipients (dose 5+)	0	0	0	1.56	0	0	0	0	0	40	4.76	0	0	20	0	0	0

LuTx recipients (dose 5-)	11.11	0	0	0	0	8.33	0	0	0	0	0	0	0	11.11	0	0	0

Table S5. Statistical analysis of RBD-specific MBC subset frequencies, related to Figure 6.

	IgM+ IgD+ resting	IgG+ DN1	IgG+ resting	IgG+ DN2
**In wt+ RBD**				
HTx recipients (dose 5+) vs HTx recipients (dose 5-)	0.382	0.445	0.584	0.698
LuTx recipients (dose 5+) vs LuTx recipients (dose 5-)	>.999	0.897	0.143	0.662
**In wt+ BA.2+ RBD**				
HTx recipients (dose 5+) vs HTx recipients (dose 5-)	0.323	0.929	0.601	0.790
LuTx recipients (dose 5+) vs LuTx recipients (dose 5-)	**0.017**	**0.049**	0.735	0.350
**In BA.2+ RBD**				
HTx recipients (dose 5+) vs HTx recipients (dose 5-)	0.338	0.170	0.694	0.086
LuTx recipients (dose 5+) vs LuTx recipients (dose 5-)	**0.030**	0.838	0.457	0.193

SUPPLEMENTAL METHODS S1

COMPUTATIONAL MODELING

To identify clinical factors associated to SARS-CoV-2 vaccine immunogenicity in solid organ transplant (SOT) recipients, unsupervised clustering was applied to a standardized four-dimensional serological feature space comprising IgG concentrations and ACE2/RBD binding inhibition capacity against wild-type (wt) and Omicron BA.2 antigens. Eight clustering workflows were evaluated by combining two algorithms—k-means and Gaussian Mixture Modelling (GMM)—across four data representations:

1. Original 4D standardized space. The four serological variables after log2 transformation of IgG concentrations and z-score scaling; no dimensionality reduction applied.
2. PCA-reduced space. Principal component analysis (PCA) applied to the 4D standardized space, retaining components that cumulatively explained ≥80% of the total variance.
3. t-SNE embedding. A non-linear dimensionality reduction that preserves local neighborhood structure but distorts global distances.
4. UMAP embedding. A non-linear method that preserves both local and, to a greater extent than t-SNE, global topological structure.

For each representation, k-means (optimal k selected by maximizing average silhouette width across k = 2–10) and GMM (optimal number of components selected by minimizing the Bayesian Information Criterion) were applied independently, yielding eight clustering workflows in total. Cluster quality was assessed using two complementary metrics: the Within-Cluster Sum of Squares (WSS), which quantifies intra-cluster compactness, and the Average Silhouette Width (ASW), which jointly measures compactness and inter-cluster separation.

Cluster selection curves.

Figure S2 shows the ASW and WSS as a function of the number of clusters k (from 2 to 10) for k-means applied to each of the four data representations. Dashed vertical lines indicate the k selected by ASW maximization. The ASW curves revealed a consistent pattern: all methods reached their maximum at k = 2, reflecting a strong primary separation within the data. Notably, the UMAP curve exhibited a markedly shallower decline from k = 2 to k = 3 (ASW = 0.577 at k = 3, compared with its maximum of ~0.588 at k = 2) relative to the Original 4D and PCA-reduced curves, suggesting that UMAP captured a secondary cluster structure at k = 3 that was not clearly resolved in non-embedding spaces. The WSS

curves for Original 4D, PCA-reduced, and UMAP all displayed a slight decrease at k = 2–3, confirming that the addition of a third cluster could provide a reduction in intra-cluster variance.

Clustering metrics across all methods.

In Figure S1 are reported the clustering of the /four z-scored immunological features using k-means and Gaussian mixture model (GMM) across the original 4D standardized space, PCA-reduced space, t-SNE embedding and UMAP embedding. Table S1 reported the number of clusters identified and the corresponding WSS and ASW for all eight clustering. Several observations emerge. First, GMM consistently produced lower ASW values than k-means across all data representations, suggesting that k-means is better suited to the structure of these data than the elliptical components fitted by GMM. Second, t-SNE-based clustering operates on a fundamentally different scale (WSS: [2300–2400] versus [200–400] for all other methods), a consequence of the severe distortion of global distances inherent to t-SNE; its WSS values are therefore not directly comparable across methods. Third, the three k-means solutions applied to Original 4D, PCA-reduced, UMAP yielded high and comparable ASW values (0.538, 0.588, and 0.577, respectively), suggesting a robust underlying data structure across these representations. Of the two solutions with the highest ASW—k-means on PCA-reduced space and k-means on UMAP—the latter achieved a lower WSS (367 versus 385), indicating greater intra-cluster compactness.

Contingency analysis: relationship between the k = 2 and k = 3 solutions.

To characterize the structural relationship between the two-cluster solution (Original 4D / PCA-reduced) and the three-cluster solution (UMAP), the cluster assignments from k-means on Original 4D (k = 2) were cross-tabulated against those from k-means on UMAP (k = 3; Table S2).

The contingency table revealed a clear hierarchical structure. Original 4D cluster 2 (n = 29) mapped almost entirely onto UMAP cluster 3 (28 of 29 subjects; 96.6%), with only one subject assigned discordantly. UMAP cluster 3 corresponded to the Low Responders (LR)—the cluster with the most distinct serological profile, characterized by the lowest IgG concentrations and lowest ACE2/RBD binding inhibition. The primary separation between LR and the remaining cohort was therefore robust and identified concordantly by all clustering methods regardless of the data representation used.

Original 4D cluster 1 (n = 88), encompassing subjects with higher antibody responses, was partitioned by UMAP into two subgroups: UMAP cluster 1 (n = 39, High Responders, HR) and UMAP cluster 2 (n = 46, Medium Responders, MR). This subdivision, not resolved by k-means in the Original 4D or

PCA-reduced spaces at k = 2, emerged at k = 3 on the UMAP embedding with a well-supported ASW of 0.577. The HR and MR groups differed significantly in IgG anti-spike concentrations (wt and BA.2) and in ACE2/RBD binding inhibition capacity (Figure 2A–D of the main manuscript), suggesting a genuine subdivision reflecting biological and clinical heterogeneity.

Selection of the clustering method

The comparative analysis of clustering workflows revealed a consistent and interpretable structure in the serological data. Across all representations, k-means outperformed GMM in terms of cluster separation, and t-SNE-based solutions proved unstable and discordant with all other methods. The non-distorting spaces (Original 4D and PCA-reduced) identified a robust primary separation between low responders and the remainder of the cohort, a structure that was preserved in the UMAP solution with 96.6% concordance. Importantly, the UMAP embedding additionally resolved a secondary partition within the high-response group, distinguishing medium from high responders — a distinction supported by statistically significant differences in serological and memory B cell outcomes (Figure 3A–D and Figure 3F-H), and by a well-maintained silhouette score (ASW = 0.577) comparable to the best value observed across all workflows. In light of these results, k-means clustering applied to the UMAP embedding with k = 3 was selected for downstream analyses, as it provided the most complete and biologically interpretable representation of immune response heterogeneity within the SOT recipient cohort.
